# Multiple Pathways To Avoid Beta Interferon Sensitivity of HIV-1 by Mutations in Capsid

**DOI:** 10.1128/JVI.00986-19

**Published:** 2019-11-13

**Authors:** Tahmina Sultana, João I. Mamede, Akatsuki Saito, Hirotaka Ode, Kyotaro Nohata, Romy Cohen, Emi E. Nakayama, Yasumasa Iwatani, Masahiro Yamashita, Thomas J. Hope, Tatsuo Shioda

**Affiliations:** a Research Institute for Microbial Diseases, Osaka University, Osaka, Japan; b Department of Cell and Developmental Biology, Northwestern University, Chicago, Illinois, USA; c Clinical Research Center, National Hospital Organization Nagoya Medical Center, Nagoya, Japan; d Aaron Diamond AIDS Research Center, New York, New York, USA; e Division of Basic Medicine, Nagoya University Graduate School of Medicine, Nagoya, Japan; University of Utah

**Keywords:** HIV-1, host factors, interferons, reverse transcription, uncoating

## Abstract

Type I interferons (IFNs), including alpha IFN (IFN-α) and IFN-β, potently suppress HIV-1 replication by upregulating IFN-stimulated genes (ISGs). The viral capsid protein (CA) partly determines the sensitivity of HIV-1 to IFNs. However, it remains to be determined whether CA-related functions, including utilization of known host factors, reverse transcription, and uncoating, affect the sensitivity of HIV-1 to IFN-mediated restriction. Recently, we identified an HIV-1 CA variant that is unusually sensitive to IFNs. This variant, called the RGDA/Q112D virus, contains multiple mutations in CA: H87R, A88G, P90D, P93A, and Q112D. To investigate how an IFN-hypersensitive virus can evolve to overcome IFN-β-mediated blocks targeting the viral capsid, we adapted the RGDA/Q112D virus in IFN-β-treated cells. We successfully isolated IFN-β-resistant viruses which contained either a single Q4R substitution or the double amino acid change G94D/G116R. These two IFN-β resistance mutations variably changed the sensitivity of CA binding to human myxovirus resistance B (MxB), cleavage and polyadenylation specificity factor 6 (CPSF6), and cyclophilin A (CypA), indicating that the observed loss of sensitivity was not due to interactions with these known host CA-interacting factors. In contrast, the two mutations apparently functioned through distinct mechanisms. The Q4R mutation dramatically accelerated the kinetics of reverse transcription and initiation of uncoating of the RGDA/Q112D virus in the presence or absence of IFN-β, whereas the G94D/G116R mutations affected reverse transcription only in the presence of IFN-β, most consistent with a mechanism of the disruption of binding to an unknown IFN-β-regulated host factor. These results suggest that HIV-1 can exploit multiple, known host factor-independent pathways to avoid IFN-β-mediated restriction by altering capsid sequences and subsequent biological properties.

**IMPORTANCE** HIV-1 infection causes robust innate immune activation in virus-infected patients. This immune activation is characterized by elevated levels of type I interferons (IFNs), which can block HIV-1 replication. Recent studies suggest that the viral capsid protein (CA) is a determinant for the sensitivity of HIV-1 to IFN-mediated restriction. Specifically, it was reported that the loss of CA interactions with CPSF6 or CypA leads to higher IFN sensitivity. However, the molecular mechanism of CA adaptation to IFN sensitivity is largely unknown. Here, we experimentally evolved an IFN-β-hypersensitive CA mutant which showed decreased binding to CPSF6 and CypA in IFN-β-treated cells. The CA mutations that emerged from this adaptation indeed conferred IFN-β resistance. Our genetic assays suggest a limited contribution of known host factors to IFN-β resistance. Strikingly, one of these mutations accelerated the kinetics of reverse transcription and uncoating. Our findings suggest that HIV-1 selected multiple, known host factor-independent pathways to avoid IFN-β-mediated restriction.

## Abstract (precis)

HIV-1 infection causes robust innate immune activation in virus-infected patients. This immune activation is characterized by elevated levels of type I interferons (IFNs), which can block HIV-1 replication. Recent studies suggest that the viral capsid protein (CA) is a determinant for the sensitivity of HIV-1 to IFN-mediated restriction. Specifically, it was reported that the loss of CA interactions with CPSF6 or CypA leads to higher IFN sensitivity. However, the molecular mechanism of CA adaptation to IFN sensitivity is largely unknown. Here, we experimentally evolved an IFN-β-hypersensitive CA mutant which showed decreased binding to CPSF6 and CypA in IFN-β-treated cells. The CA mutations that emerged from this adaptation indeed conferred IFN-β resistance. Our genetic assays suggest a limited contribution of known host factors to IFN-β resistance. Strikingly, one of these mutations accelerated the kinetics of reverse transcription and uncoating. Our findings suggest that HIV-1 selected multiple, known host factor-independent pathways to avoid IFN-β-mediated restriction.

## INTRODUCTION

It is well established that type I interferons (IFNs) suppress a wide range of viruses, including HIV-1, by upregulating interferon-stimulated genes (ISGs). Although the molecular mechanisms of the type I IFN-mediated suppression of HIV-1 had been unclear for a long time, recent studies identified several interferon-inducible host factors which suppress HIV-1 replication (1–7). These include SAM domain- and HD domain-containing protein 1 (SAMHD1), human myxovirus resistance B (MxB; also known as Mx2), and bone marrow stromal cell antigen 2 (BST-2; also known as tetherin, CD317, or HM1.24).

Multiple viral elements are involved in the type I IFN sensitivity of HIV-1 (8, 9). Importantly, recent studies suggested that the viral capsid protein (CA) sequence affected type I IFN sensitivity (10, 11). CA is a multifunctional protein that orchestrates several steps of HIV-1 infection, including reverse transcription (12–15), nuclear entry (16–20), and integration of viral DNA into host cell chromatin (18, 21). Multiple host factors, including cyclophilin A (CypA) and cleavage and polyadenylation specificity factor 6 (CPSF6), are also involved in promoting or inhibiting HIV-1 infection by interacting with CA (22–26). Interestingly, these host proteins affect type I IFN sensitivity. Specifically, it was reported that the CypA binding-deficient CA mutant (the P90A mutant) and the CPSF6 binding-deficient CA mutant (the N74D and A105T mutant) are more sensitive than wild-type (WT) CA to IFN alpha (IFN-α) in monocyte-derived THP-1 cells (10). This phenotype is of interest since these CA mutants were shown to be resistant to MxB, a type I IFN-inducible, potent anti-HIV-1 host factor whose antiviral effect is influenced by viral CA (11, 27–30).

Another prominent host molecule that targets the viral capsid is a group of the TRIM5 proteins, including TRIM5α and TRIMCyp (31–33). TRIM5 proteins potently restrict HIV-1 in certain monkey cells and limit the interspecies transmission of HIV-1 (33). TRIMCyp is a naturally occurring fusion protein between TRIM5α and CypA in certain monkey species (32, 34, 35). To establish a macaque model for HIV-1 infection, we and others constructed HIV-1 derivatives capable of establishing productive infection in monkey cells by manipulating the CA sequence, a major determinant for species specificity (36–39). We recently reported that one mutant virus, called the RGDA/Q112D virus, which contains the H87R, A88G, P90D, P93A, and Q112D changes in CA, was highly resistant to cynomolgus monkey (CM) TRIMCyp (40). An interesting property of this RGDA/Q112D virus is its behavior in cells coinfected with Sendai virus (SeV). Coinfection of target cells with SeV enhanced the infectivity of the RGDA/Q112D virus but not that of the wild-type (WT) virus. As SeV attenuates a type I IFN-induced antiviral state (41), we hypothesized that the RGDA/Q112D mutant is hypersensitive to type I IFN. A potential mechanistic link to this observation is the loss of capsid binding to CypA, a molecule known to affect HIV-1 sensitivity to type I IFN (10).

In this study, we used this RGDA/Q112D virus as a tool to study the interplay between HIV-1 CA and type I IFN-mediated restriction. We first demonstrate that the RGDA/Q112D virus is indeed highly sensitive to IFN-β in Jurkat T cells. We next performed adaptation of the RGDA/Q112D virus in IFN-β-treated Jurkat cells to ask how a highly IFN-β-sensitive virus evolves to overcome capsid-targeting restriction by type I IFN. We found that a single Q4R mutation or the double substitutions G94D/G116R in CA emerged during adaptation and conferred IFN-β resistance upon transfer to the parental RGDA/Q112D virus. Importantly, the Q4R mutation accelerated the kinetics of the completion of reverse transcription and the initiation of uncoating of the RGDA/Q112D virus, whereas the reverse transcription kinetics of the RGDA/Q112D virus were also accelerated by the G94D/G116R mutations. These results reveal multiple mutational escape pathways, which are independent of known host factors, to avoid IFN-β-mediated restriction targeting the viral capsid.

## RESULTS

### A cyclophilin A binding-deficient capsid mutant displays enhanced IFN-β sensitivity in T cells.

**FIG 1:**
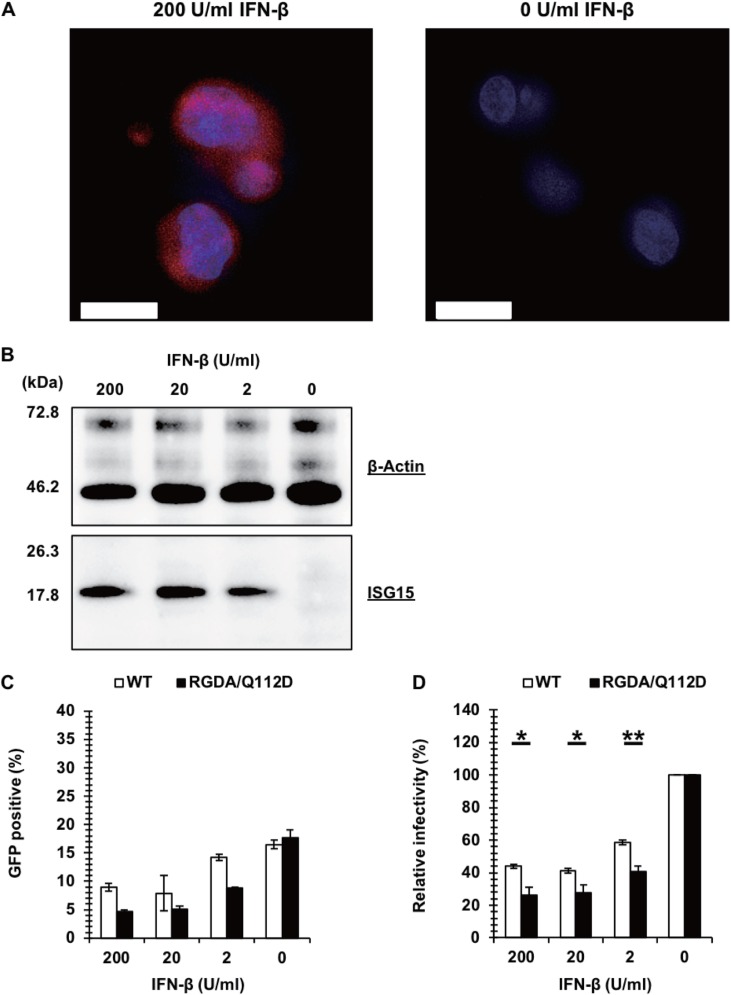
A cyclophilin A binding-deficient capsid mutant, the RGDA/Q112D virus, is hypersensitive to IFN-β in T cells. (A) Jurkat cells treated with 200 U per ml of IFN-β or left untreated were examined for the induction of ISG15 (red, ISG15 monoclonal antibody; blue, Hoechst dye). Bars, 10 μm. (B) Expression level of ISG15 in Jurkat cells treated with 200, 20, 2, or 0 U per ml of IFN-β. Western blots of cell lysates extracted from Jurkat cells were probed with an anti-ISG15 antibody (bottom) or an anti-β-actin antibody (top). The positions of the molecular weight markers are shown on the left side. (C) Jurkat cells treated with 200 U, 20 U, 2 U, or 0 U per ml of IFN-β were infected with VSV-G-pseudotyped GFP reporter viruses. The level of GFP expression was determined at 2 days after infection. One representative result of at least three independent experiments is shown, with error bars denoting the standard deviation (SD) of the mean of triplicate measurements. (D) The relative IFN-β sensitivity (compared with that for untreated cells [in percent]) was calculated by dividing the percentage of GFP-positive cells among IFN-β-treated cells by that among untreated cells. The mean from three independent experiments is shown, with error bars denoting the standard error of the mean (SEM). **, *P *< 0.01; *, *P *< 0.05.

We first examined the IFN-β sensitivity of the RGDA/Q112D virus (40), since the P90A mutation in CA, which results in a CypA binding-deficient mutant (42, 43), has been shown to increase the sensitivity to IFN-α-mediated inhibition (10). We used Jurkat cells, a T cell line, since T cells are the major target for HIV-1 replication. Jurkat cells were treated with increasing concentrations (0, 2, 20, and 200 U per ml) of IFN-β. Protein expression of ISG15, a representative ISG (44), was readily observed in IFN-β-treated cells with immunofluorescence staining (Fig. 1A) and Western blot analyses (Fig. 1B), suggesting that this cell line had an intact cascade of type I interferon signaling. To test the IFN-β sensitivity of the RGDA/Q112D virus, Jurkat cells treated with IFN-β or left untreated were challenged with green fluorescent protein (GFP)-expressing viruses. This is consistent with the hypothesis that the RGDA/Q112D virus is more sensitive to IFN-β than the WT virus, and we found a significantly decreased infectivity of the RGDA/Q112D virus in IFN-β-treated cells compared with that of the WT virus (Fig. 1C and D).

### Adaptation of the RGDA/Q112D virus in IFN-β-treated T cells.

**FIG 2:**
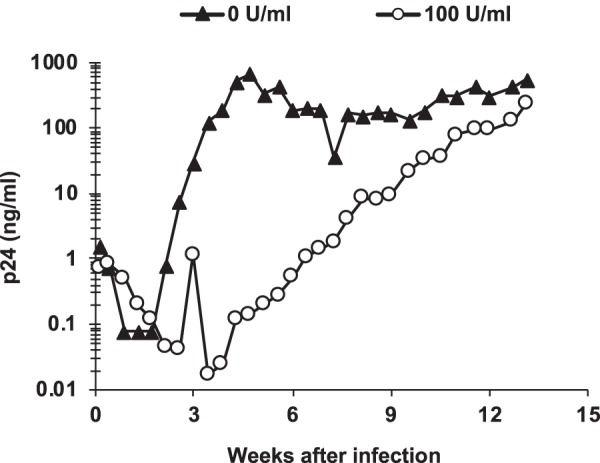
Adaptation of the RGDA/Q112D virus in IFN-β-treated Jurkat cells. Jurkat cells were treated with 100 U per ml of IFN-β or left untreated for 6 h. Cells were infected with 100 ng (p24) of the NL-VifS virus encoding the RGDA/Q112D mutations. The viral titers in the culture supernatant were measured periodically using a p24 ELISA kit.

To determine if the RGDA/Q112D virus could develop IFN-β resistance, we adapted the virus through long-term culture in IFN-β-treated Jurkat and MT4 cells. IFN-β-treated Jurkat and MT4 cells were infected with the RGDA/Q112D virus, and viral replication was monitored during an extended period of culture. While the RGDA/Q112D virus was unable to escape IFN-β inhibition in MT4 cells (data not shown), the RGDA/Q112D virus started to replicate in IFN-β-treated Jurkat cells approximately 5 weeks after infection (Fig. 2). By 13 weeks, the emerging IFN-β-resistant virus produced p24 at levels comparable to those produced by untreated Jurkat cells. We were interested in the evolution of the CA sequences and their impact on IFN-β sensitivity and PCR amplified CA regions from the genomic DNA of infected cells after 92 days in culture to identify potential changes facilitating resistance. Sequence analysis of 20 clones of TOPO plasmids revealed the presence of three types of CA sequences in these clones: the RGDA/Q112D+Q4R, RGDA/Q112D+G94D, and RGDA/Q112D+G94D/G116R clones. It should be noted that the G116R mutation was found only in the clones that also harbored the G94D mutation. Moreover, only the G116R mutation was associated with the G94D mutation. The frequency of the RGDA/Q112D+Q4R, RGDA/Q112D+G94D, and RGDA/Q112D+G94D/G116R sequences was 30%, 5%, and 10%, respectively. We observed that other clones (55%) encoded the RGDA/Q112D mutations, meaning that any substitution or reversion to the WT sequence in viruses with the original five mutations (H87R, A88G, P90D, P93A, and Q112D) was not detected.

### CA mutations in the adapted virus confer IFN-β resistance to the RGDA/Q112D virus.

**FIG 3:**
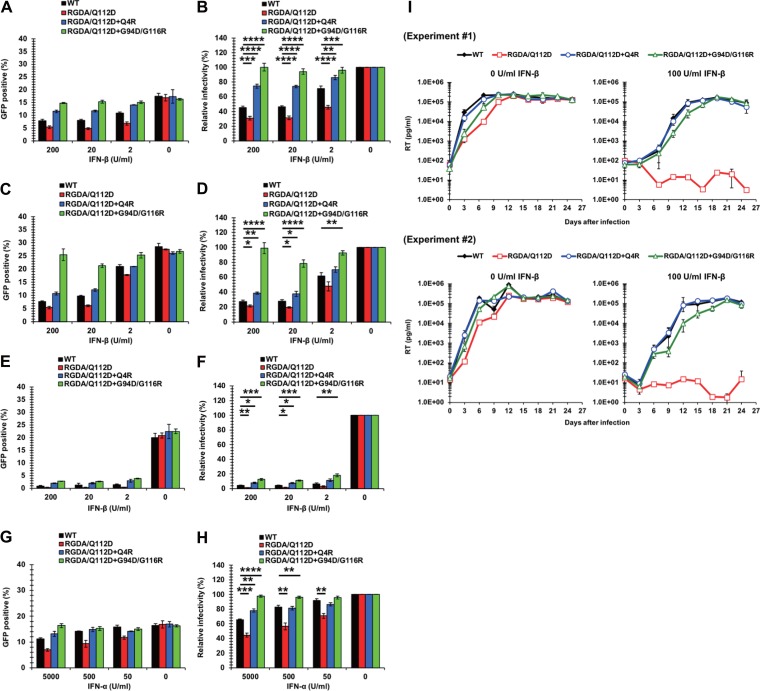
CA mutations in the adapted viruses confer IFN-β resistance on the RGDA/Q112D virus. (A) Jurkat cells were treated with 200, 20, 2, or 0 U per ml of IFN-β for 16 h prior to infection. Cells were infected with VSV-G-pseudotyped HIV-1 isolates encoding GFP. The percentage of GFP-positive cells was determined at 2 days after infection. One representative result of at least three independent experiments is shown, with error bars denoting the standard deviation (SD) of the mean of triplicate measurements. (B) The relative IFN-β sensitivity (compared with that in untreated cells [in percent]) was calculated by dividing the percentage of GFP-positive cells among the IFN-β-treated cells by the percentage of GFP-positive cells among untreated cells. The mean from seven independent experiments is shown, with error bars denoting the standard error of the mean (SEM). ****, *P *< 0.0001; ***, *P *< 0.001; **, *P *< 0.01. (C) MT4 cells were treated with 200, 20, 2, or 0 U per ml of IFN-β for 16 h prior to infection. Cells were infected with VSV-G-pseudotyped HIV-1 isolates encoding the GFP reporter gene. The percentage of GFP-positive cells was determined at 2 days after infection. One representative result of at least three independent experiments is shown, with error bars denoting the standard deviation (SD) of the mean of triplicate measurements. (D) The relative IFN-β sensitivity (compared with that in untreated cells [in percent]) was calculated by dividing the percentage of GFP-positive cells among IFN-β-treated cells by the percentage of GFP-positive cells among untreated cells. The mean from four independent experiments is shown, with error bars denoting the standard error of the mean (SEM). ****, *P *< 0.0001; **, *P *< 0.01; *, *P *< 0.05. (E) THP-1 cells were treated with 200, 20, 2, or 0 U per ml of IFN-β for 16 h prior to infection. Cells were infected with VSV-G-pseudotyped HIV-1 isolates encoding the GFP reporter gene. The percentage of GFP-positive cells was determined at 2 days after infection. One representative result of at least three independent experiments is shown, with error bars denoting the standard deviation (SD) of the mean of triplicate measurements. (F) The relative IFN-β sensitivity (compared with that in untreated cells [in percent]) was calculated by dividing the percentage of GFP-positive cells among IFN-β-treated cells by the percentage of GFP-positive cells among untreated cells. The mean from four independent experiments is shown, with error bars denoting the standard error of the mean (SEM). ***, *P *< 0.001; **, *P *< 0.01; *, *P *< 0.05. (G) Jurkat cells were treated with 5,000, 500, 50, or 0 U per ml of IFN-α for 16 h prior to infection. Cells were infected with VSV-G-pseudotyped HIV-1 isolates encoding the GFP reporter gene. The percentage of GFP-positive cells was determined at 2 days after infection. One representative result of at least three independent experiments is shown, with error bars denoting the standard deviation (SD) of the mean of triplicate measurements. (H) The relative IFN-α sensitivity (compared with that in untreated cells [in percent]) was calculated by dividing the percentage of GFP-positive cells among IFN-α-treated cells by the percentage of GFP-positive cells among untreated cells. The mean of five independent experiments is shown, with error bars denoting the standard error of the mean (SEM). ****, *P *< 0.0001; ***, *P *< 0.001; **, *P *< 0.01. (I) Jurkat cells were left untreated or treated with 100 U per ml of IFN-β for 16 h prior to infection. Cells were challenged with NL4-3 viruses normalized to 1,000 pg per ml. Half of the culture medium was replaced with fresh medium containing 0 or 100 U per ml of IFN-β every 3 days, and the concentration of reverse transcriptase (RT) in the culture supernatant was quantified by the SG-PERT assay. The results of two independent experiments are shown, with error bars denoting the standard deviation (SD) of the mean of triplicate measurements.

To examine the contribution of CA mutations found in the adapted viruses to IFN-β sensitivity, we introduced the individual mutations into a ΔEnv molecular clone carrying a GFP reporter gene. Jurkat cells were treated with IFN-β or left untreated and then challenged with vesicular stomatitis virus G glycoprotein (VSV-G)-pseudotyped, GFP-encoding viruses. The RGDA/Q112D+Q4R virus was more resistant to IFN-β than the RGDA/Q112D or the WT virus at all IFN-β concentrations (Fig. 3A and B). Notably, the RGDA/Q112D+G94D/G116R virus was completely IFN-β resistant even to the highest concentration (200 U per ml) of IFN-β. The IFN-β sensitivity of the RGDA/Q112D+G94D virus was not tested because it was not infectious. The individual impacts of these CA mutations on IFN-β resistance of the RGDA/Q112D virus were not specific to Jurkat cells since we observed a similar phenotype in another CD4-positive T cell line, MT4 cells (Fig. 3C and D). Furthermore, the RGDA/Q112D+Q4R and RGDA/Q112D+G94D/G116R viruses showed partial but significant IFN-β resistance in the monocytic cell line THP-1 (Fig. 3E and F). The significant IFN resistance of these CA mutations was observed with IFN-α (Fig. 3G and H), in addition to IFN-β.

We extended our observations to examine the impact of the identified CA resistance mutations in the context of the replication-competent pNL4-3 isolate and examined the viral replication of these derivatives in untreated and IFN-β-treated Jurkat cells. We observed the robust replication of the RGDA/Q112D virus in untreated cells (Fig. 3I, left), but, as expected, its replication was severely suppressed in IFN-β-treated cells up to 4 weeks after infection (Fig. 3I, right). Notably, RGDA/Q112D viruses harboring Q4R or G94D/G116R mutations efficiently replicated in both untreated and IFN-β-treated cells (Fig. 3I), suggesting that either the Q4R mutation or the G94D/G116R mutation is sufficient to confer IFN-β resistance to the RGDA/Q112D background during spreading infection. These results demonstrate that the Q4R and G94D/G116R mutations in CA in adapted viruses are sufficient to provide IFN-β resistance to RGDA/Q112D viruses.

**FIG 4:**
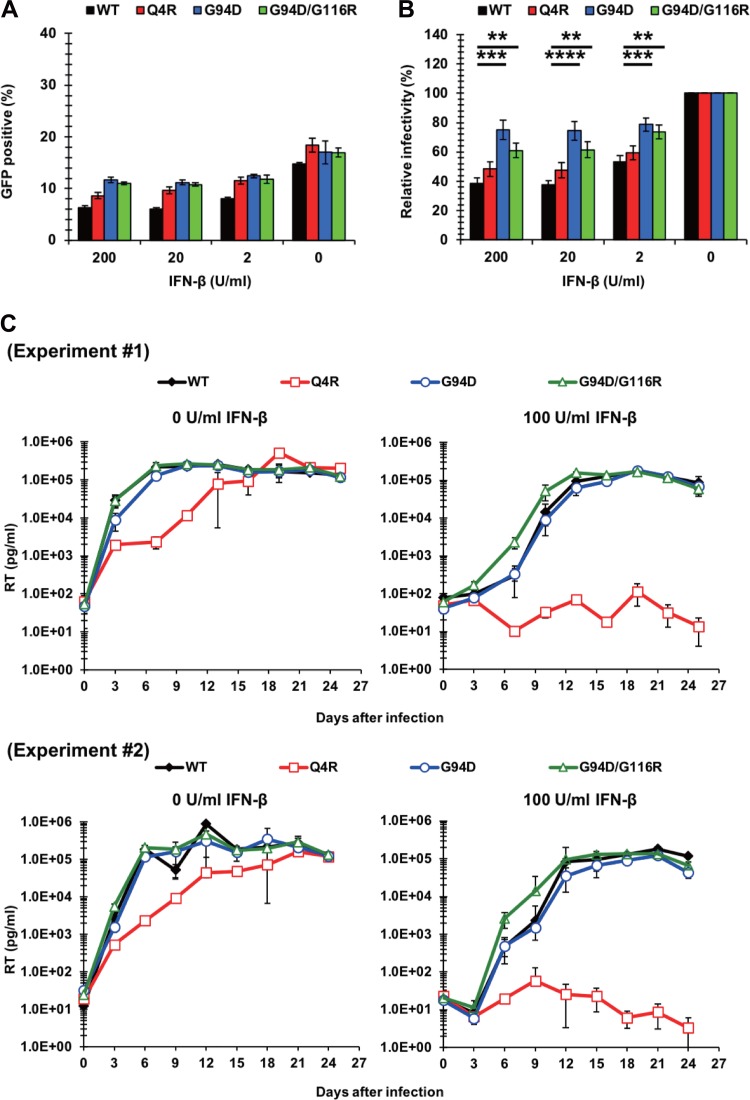
The G94/G116R mutation confers IFN-β resistance to the WT virus. (A) Jurkat cells were treated with 200, 20, 2, or 0 U per ml of IFN-β for 16 h prior to infection. Cells were infected with VSV-G-pseudotyped HIV-1 isolates encoding the GFP reporter gene. The percentage of GFP-positive cells was determined at 2 days after infection. One representative result of at least three independent experiments is shown, with error bars denoting the standard deviation (SD) of the mean of triplicate measurements. (B) The relative IFN-β sensitivity (compared with that in untreated cells [in percent]) was calculated at 2 days after infection by dividing the percentage of GFP-positive cells among IFN-β-treated cells by the percentage of GFP-positive cells among untreated cells. The mean of nine independent experiments is shown, with error bars denoting the standard error of the mean (SEM). ****, *P *< 0.0001; ***, *P *< 0.001; **, *P *< 0.01. (C) Jurkat cells were left untreated or treated with 100 U per ml of IFN-β for 16 h prior to infection. Cells were challenged with NL4-3 viruses normalized to 1,000 pg per ml. Half of the culture medium was replaced with fresh medium every 3 days, and the concentration of reverse transcriptase (RT) in the culture supernatant was quantified by the SG-PERT assay. The results of two independent experiments are shown, with error bars denoting the standard deviation (SD) of the mean of triplicate measurements. Note that the values for the WT virus in each experiment were also used in Fig. 3I.

To further characterize the Q4R and G94D/G116R mutations, we examined their impact on IFN-β resistance in the context of WT CA. In the context of VSV-G-pseudotyped viruses that had undergone a single round of infection, the G94D/G116R mutation and the G94D mutation conferred IFN-β resistance to the WT virus, while the Q4R mutation did not affect IFN-β sensitivity in this WT CA context (Fig. 4A and B). We further tested the IFN-β sensitivity of NL4-3 CA mutants harboring the Q4R and G94D/G116R mutations in a multiround replication assay by using untreated Jurkat cells or cells treated with 100 U per ml of IFN-β. We observed the robust replication of all the viruses in untreated cells. However, we noted that the NL4-3 Q4R virus showed a slight delay in viral replication compared to the other viruses (Fig. 4C, left). This difference became more obvious in IFN-β-treated cells, where the replication of the NL4-3 Q4R virus was severely attenuated (Fig. 4C, right). These results reveal that the Q4R mutation specifically conferred IFN-β resistance in the context of the RGDA/Q112D virus, whereas the G94D/G116R mutations conferred IFN-β resistance to both the RGDA/Q112D virus and the WT virus.

### The Q4R mutation sensitizes the RGDA/Q112D virus to MxB-mediated restriction despite conferring IFN-β resistance.

**FIG 5:**
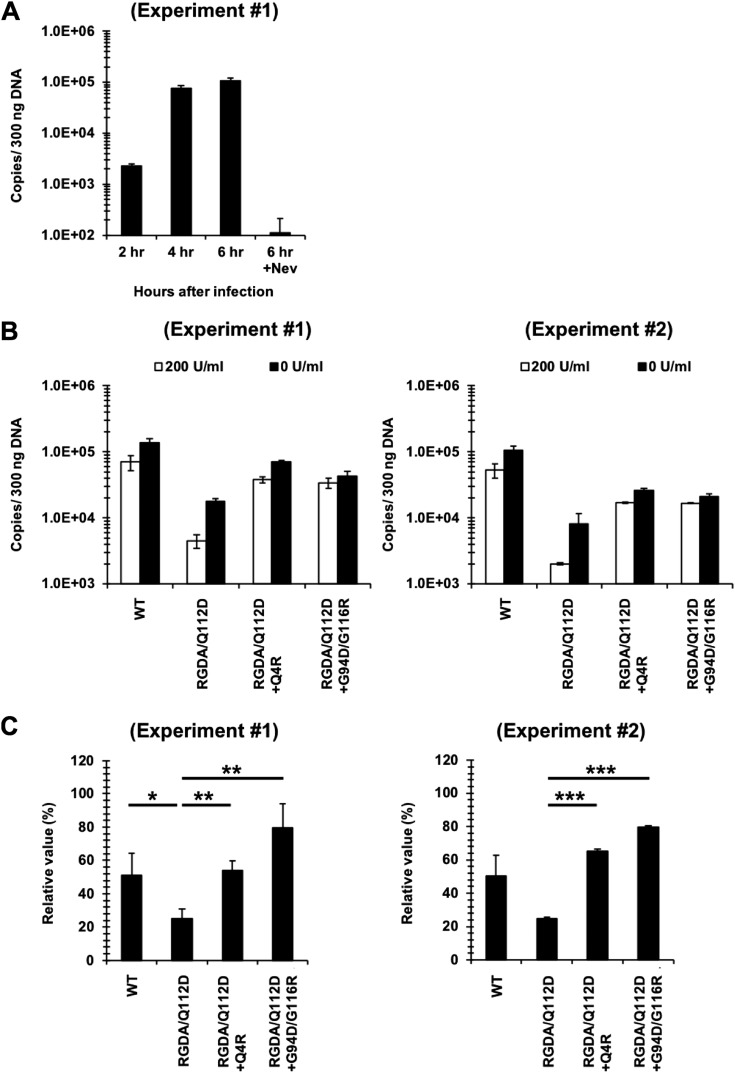
Steps at or before the second-strand transfer of reverse transcription of the RGDA/Q112D virus were suppressed by IFN-β. (A) Untreated Jurkat cells were infected with the WT virus. DNA extracted at 2, 4, and 6 h after infection was used for PCR to quantify the second-strand transfer products of reverse transcription. Results are shown as the number of copies normalized to the DNA concentrations (number of copies per 300 ng DNA). Cells treated with 5 μM nevirapine (Nev) served as a negative control. Representative data from one of two independent experiments are shown, with error bars denoting the standard deviation (SD) of the mean of duplicate measurements. (B) Jurkat cells treated with 0 or 200 U per ml of IFN-β were infected with the CA mutants. DNA extracted at 6 h after infection was used for PCR to quantify the second-strand transfer products of reverse transcription. Results are shown as the number of copies normalized to the DNA concentrations (number of copies per 300 ng DNA). The results of two independent experiments are shown, with error bars denoting the standard deviation (SD) of the mean of triplicate measurements. (C) The suppressive effect of IFN-β on the generation of second-strand transfer products of reverse transcription at 6 h after infection was evaluated by dividing the copy number of the second-strand transfer products in the presence of 200 U per ml of IFN-β by the copy number in the absence of IFN-β. The results of two independent experiments are shown, with error bars denoting the standard deviation (SD) of the mean of triplicate measurements. ***, *P *< 0.001; **, *P *< 0.01; *, *P *< 0.05.

Next, we studied the mechanisms of the IFN-β sensitivity of the parental and adapted viruses. First, we tested whether IFN-β affected the levels of HIV-1 reverse transcription. We quantified the levels of the second-strand transfer products in Jurkat cells infected with VSV-G-pseudotyped viruses with or without IFN-β. The levels of second-strand transfer products in cells infected with the WT virus without IFN-β reached a plateau at 4 to 6 h after infection (Fig. 5A). We therefore compared the levels of second-strand transfer products at 6 h after infection. Figures 5B and C show that the levels of the second-strand transfer products of the RGDA/Q112D virus were more strongly suppressed by IFN-β than those of the WT virus and that the Q4R and G94D/G116R substitutions abolished the suppressive effects of the RGDA/Q112D substitutions in the presence of IFN-β. These results indicate that a step or steps occurring until the completion of second-strand transfer of reverse transcription by the RGDA/Q112D virus were at least one of the targets for IFN-β.

**FIG 6:**
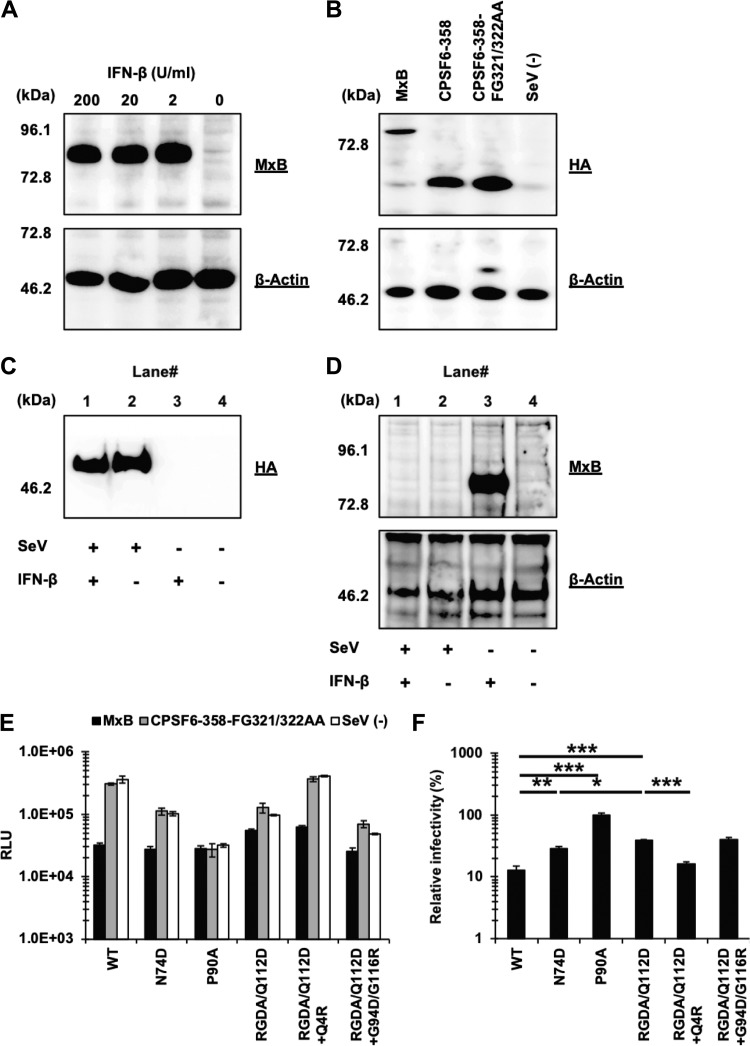
The Q4R mutation sensitizes the RGDA/Q112D virus to MxB restriction despite conferring IFN-β resistance. (A) Expression level of MxB in Jurkat cells treated with 200, 20, 2, or 0 U per ml of IFN-β. Western blots of cell lysates extracted from IFN-β-treated cells were probed with an anti-MxB antibody (top) or an anti-β-actin antibody (bottom). The positions of the molecular weight markers are shown on the left side. (B) The expression level of HA-tagged MxB, CPSF6-358, and CPSF6-358-FG321/322AA (control) in SeV-infected MT4 cells was evaluated using a rat anti-HA monoclonal antibody (top). The membrane was reprobed with an anti-β-actin antibody (bottom). The positions of the molecular weight markers are shown on the left side. (C) The expression level of HA-tagged CPSF6-358-FG321/322AA in SeV-infected MT4 cells was evaluated using a rat anti-HA monoclonal antibody. The position of the molecular weight marker is shown on the left side. (D) Expression level of MxB in MT4 cells infected with SeV expressing CPSF6-358-FG321/322AA in the presence of either 0 or 200 per ml IFN-β. Cell lysates were probed with an anti-MxB antibody (top) or an anti-β-actin antibody (bottom). The positions of the molecular weight markers are shown on the left side. (E) MT4 cells expressing MxB or CPSF6-358-FG321/322AA or SeV^−^ cells were superinfected with reverse transcriptase-normalized VSV-G-pseudotyped HIV-1 isolates encoding the luciferase reporter gene. The RLU were determined at 2 days after infection. One representative result of at least three independent experiments is shown, with error bars denoting the standard deviation (SD) of the mean of triplicate measurements. (F) The degree of sensitivity to MxB was calculated by dividing the RLU of each virus in the presence of MxB by those in the presence of CPSF6-358-FG321/322AA (control). The mean from three independent experiments is shown, with error bars denoting the standard error of the mean (SEM). ***, *P *< 0.001; **, *P *< 0.01; *, *P *< 0.05.

We then tested the contribution of MxB to the IFN-β sensitivity of these viruses, since MxB is an IFN-β-inducible (Fig. 6A), potent host factor (27, 30) suppressing an early step of HIV-1 infection. We used a Sendai virus (SeV) expression vector for expression of hemagglutinin (HA)-tagged MxB in MT4 cells (Fig. 6B). As a control, we included a mutant of CPSF6-358, CPSF6-358-FG321/322AA, which does not bind to HIV-1 CA (26, 45, 46), in addition to SeV-negative (SeV^−^) mock-infected cells. Since SeV stocks containing a high content of defective interfering (DI) particles were reported to stimulate ISGs in infected human lymphoblastoid cells (47), we evaluated whether our recombinant SeV induced the expression of endogenous MxB in MT4 cells. The result showed that the SeV-expressing CPSF6-358-FG321/322AA mutant (Fig. 6C) did not induce the expression of endogenous MxB (Fig. 6D, lane 2). Furthermore, preinfection of cells with SeV canceled the expression of endogenous MxB upon IFN-β treatment of the cells (Fig. 6D, lane 1). This finding agrees with previous findings that SeV neutralizes a type I IFN-induced antiviral state by its C and/or V protein (41, 48, 49). We concluded that our SeV system could probe interactions between host factors and CA mutants in the absence of endogenous MxB expression.

MT4 cells ectopically overexpressing MxB blocked the infection of the WT virus (Fig. 6E and F). In agreement with previous reports (30), the N74D and P90A viruses were more resistant to MxB than the WT virus (Fig. 6F). The RGDA/Q112D virus was even more resistant to MxB than the N74D virus (38.5% versus 28.9%). Thus, there appeared to be no correlation between sensitivity to IFN-β and MxB.

Examination of adapted viruses also failed to show a correlation between sensitivity to IFN-β and MxB. The Q4R mutation enhanced the sensitivity of the RGDA/Q112D virus to MxB restriction, despite the fact that the Q4R mutation conferred IFN-β resistance to the RGDA/Q112D virus (Fig. 3 and Table 1). The G94D/G116R mutations did not significantly affect the sensitivity of the RGDA/Q112D virus to MxB, as we observed no difference in sensitivity to MxB between the RGDA/Q112D virus and the RGDA/Q112D+G94D/G116R virus (38.5% versus 40.2%). These observations suggest a limited role of MxB in the IFN-β sensitivity of the RGDA/Q112D virus.

**TABLE 1:** Phenotypes of CA mutants

Phenotype	Result for the following virus^*a*^:			
	WT	RGDA/Q112D mutant	RGDA/Q112D+Q4R mutant	RGDA/Q112D+G94D/G116R mutant
IFN-β resistance	+++	+	++++	++++
Sensitivity to MxB	+++	++	+++	++
Sensitivity to CPSF6-358	+++	+	+++	+/−
CypA binding	+++	−	−	−
Kinetics of reverse transcription (without IFN-β)	+++	+++	+++++	+++
Kinetics of reverse transcription (with IFN-β)	+++	+	+++++	++
Initiation of uncoating (without IFN-β)	+++	+++	+++++	+++
Initiation of uncoating (with IFN-β)	+++	+++	+++++	+++

a >+++, higher or faster than that for the WT virus; +++, comparable to that for the WT virus; <+++, lower or slower than that for the WT virus.

### Limited contributions of CPSF6 and CypA for IFN-β resistance of the RGDA/Q112D virus harboring the Q4R or G94D/G116R mutations.

**FIG 7:**
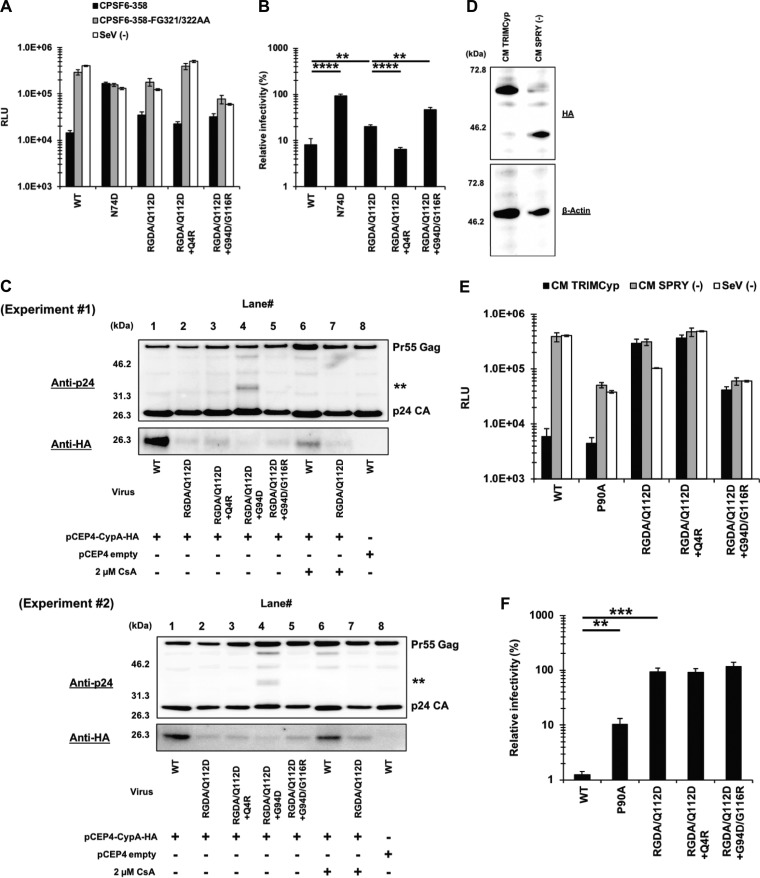
Limited contributions of CPSF6 and CypA to the IFN-β resistance of the RGDA/Q112D virus harboring the Q4R mutation or the G94D/G116R mutations. (A) MT4 cells expressing CPSF6-358 or CPSF6-358-FG321/322AA or SeV^−^ cells were superinfected with reverse transcriptase-normalized VSV-G-pseudotyped HIV-1 isolates encoding the luciferase reporter gene. The RLU were determined at 2 days after infection. One representative result of at least three independent experiments is shown, with error bars denoting the standard deviation (SD) of the mean of triplicate measurements. (B) The degree of CPSF6-358 sensitivity was calculated by dividing the RLU of each virus in the presence of CPSF6-358 by those in the presence of CPSF6-358-FG321/322AA (control). The mean from six independent experiments is shown, with error bars denoting the standard error of the mean (SEM). ****, *P *< 0.0001; **, *P *< 0.01. (C) HEK293T cells were cotransfected with pNL4-3 plasmids along with a pCEP4 vector encoding HA-tagged human CypA. Pelleted virions were subjected to Western blot analysis using a rat anti-HA monoclonal antibody (bottom) and mouse anti-p24 antibody (top). The positions of the molecular weight markers are shown on the left side. The results of two independent experiments are shown. (D) The expression levels of HA-tagged CM TRIMCyp and CM SPRY^−^ (control) in SeV-infected MT4 cells were determined using a rat anti-HA monoclonal antibody (top). The membrane was reprobed with the anti-β-actin antibody (bottom). The positions of the molecular weight markers are shown on the left side. (E) MT4 cells expressing CM TRIMCyp or CM SPRY^−^ or SeV^−^ cells were superinfected with reverse transcriptase-normalized VSV-G-pseudotyped HIV-1 isolates encoding the luciferase reporter gene. The RLU were determined at 2 days after infection. One representative result of at least three independent experiments is shown, with error bars denoting the standard deviation (SD) of the mean of triplicate measurements. (F) The degree of interaction was calculated by dividing the RLU of each virus in the presence of CM TRIMCyp by those in the presence of CM SPRY^−^. The mean from five independent experiments is shown, with error bars denoting the standard error of the mean (SEM). ***, *P *< 0.001; **, *P *< 0.01.

Another potential mechanism that modulates the sensitivity to capsid-targeting machinery that is induced by type I IFN is the CPSF6 and/or CypA association with CA. A previous study showed that a CPSF6 binding-deficient CA mutant (the N74D mutant) exhibits a higher degree of IFN-β sensitivity than the WT virus (10). To examine the interaction of the viral capsids with CPSF6, we performed an infection-based assay using a truncated version of CPSF6 (CPSF6-358), which blocks viral infection through CA interactions (25, 50). Previous studies showed that the CPSF6-358 sensitivity of CA mutants generally correlates with *in vitro* protein binding between CA and a CPSF6 peptide (26, 50–53). We used an SeV vector to express HA-tagged CPSF6-358 in MT4 cells (Fig. 6B). Cells infected with an SeV-expressing CPSF6-358-FG321/322AA mutant, in addition to mock-infected cells, served as negative controls. Infection of the WT virus was highly restricted in CPSF6-358-expressing cells compared to that in CPSF6-358-FG321/322AA-expressing or SeV^−^ cells (Fig. 7A). In contrast, infection of the N74D virus was not affected by CPSF6-358 (Fig. 7A and B). These findings validate those of our experimental assay. We found that, like its WT counterpart, the RGDA/Q112D virus was blocked by CPSF6-358. However, the relative infectivity of the RGDA/Q112D virus in CPSF6-358-expressing cells was not as low as that of the WT virus. Although the difference was rather small (20.1% versus 8.1% for the RGDA/Q112D virus and the WT virus, respectively), the difference was statistically significant (*P *< 0.01).

Next, we examined the sensitivity of the adapted variants to CPSF6-358-mediated restriction. Importantly, the relative infectivity of the RGDA/Q112D+Q4R virus in CPSF6-358-expressing cells was lower than that of the RGDA/Q112D virus (6.5% versus 20.1%, *P *< 0.0001). The relative infectivity of the RGDA/Q112D+Q4R virus in CPSF6-358-expressing cells was comparable to that of the WT virus (6.5% versus 8.1%). In contrast, the G94D/G116R mutations decreased the CPSF6-358 sensitivity of the RGDA/Q112D virus (20.1% versus 46.6% for the relative infectivity of the RGDA/Q112D virus and the RGDA/Q112D+G94D/G116R virus in CPSF6-358-expressing cells, respectively; *P *< 0.01). These results suggest that the Q4R mutation specifically enhanced the CPSF6-358 sensitivity of the RGDA/Q112D virus.

Finally, we investigated how CA mutations in IFN-β-resistant viruses affected CypA binding and determined the levels of CypA within isolated viral particles. Western blot analysis demonstrated that CypA was efficiently incorporated into the virion of the WT virus and that treatment of transfected cells with cyclosporine (CsA) decreased the incorporation of CypA into the virion (Fig. 7C, lanes 1 and 6). In contrast, the RGDA/Q112D viral particles contained marginal amount of CypA (Fig. 7C, lane 2). We observed a similar phenotype with the additional mutations in RGDA/Q112D+Q4R and RGDA/Q112D+G94D/G116R viral particles. A complementary assay using cynomolgus monkey (CM) TRIMCyp supported these observations, as the RGDA/Q112D viruses harboring the Q4R or G94D/G116R mutations were completely resistant to the antiviral activity of the CM TRIMCyp protein (Fig. 7D to F), indicating that these mutations did not restore the CypA binding of the RGDA/Q112D virus. These observations suggest that intact binding with both CPSF6 and CypA may not be necessary for evasion of the RGDA/Q112D virus from IFN-β-mediated inhibition.

### The Q4R mutation accelerates the kinetics of completion of reverse transcription of the RGDA/Q112D virus.

**FIG 8:**
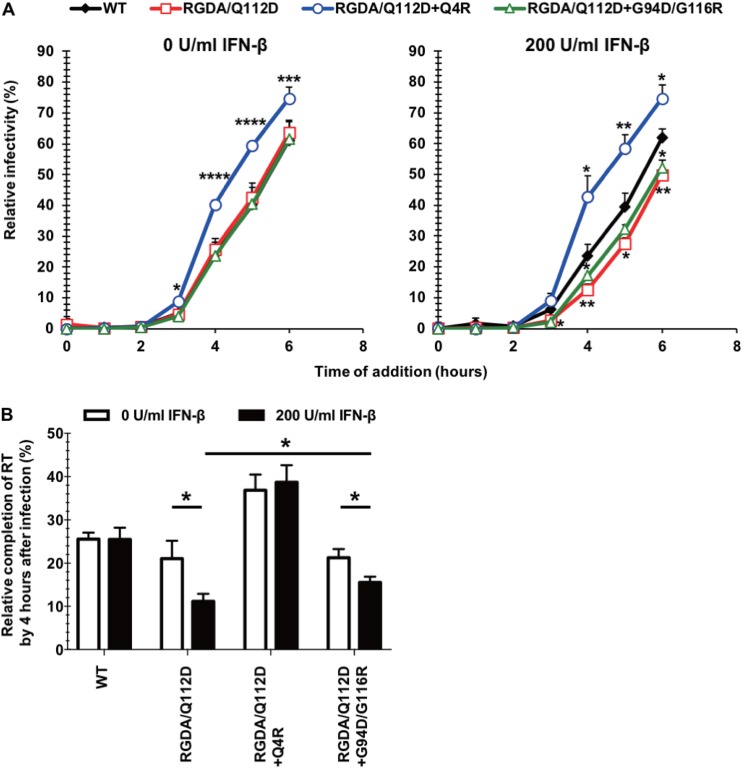
The Q4R mutation accelerates the kinetics of reverse transcription of the RGDA/Q112D virus. (A) Jurkat cells were treated with 0 or 200 U per ml of IFN-β for 16 h prior to infection. Cells were infected with VSV-G-pseudotyped HIV-1 isolates encoding the GFP reporter gene. The reverse transcriptase inhibitor nevirapine was added at the indicated time after infection. The percentage of GFP-positive cells was determined at 2 days after infection. Relative infectivity (compared with that for cells not treated with nevirapine [in percent]) was calculated by dividing the percentage of GFP-positive cells among nevirapine-treated cells by the percentage of GFP-positive cells among untreated cells. ****, *P *< 0.0001; ***, *P *< 0.001; **, *P *< 0.01; *, *P *< 0.05. (B) The influence of IFN-β treatment on reverse transcription (RT) kinetics was analyzed by using the values presented in panel A. The mean values of the relative infectivity of cells treated with nevirapine at 4 h after infection from three independent experiments are shown with standard errors of the mean (SEM). *, *P *< 0.05.

CA has pivotal roles for early events of HIV-1 replication, including reverse transcription and uncoating. We began to investigate the impact of the Q4R and G94D/G116R mutations on these events in the RGDA/Q112D context. To examine the kinetics of reverse transcription in the different derivatives, we performed a time-of-addition assay using the reverse transcriptase inhibitor nevirapine (14, 54, 55). We observed that the temporal sensitivity of the nevirapine inhibitory effects differed among the viruses examined. Specifically, the RGDA/Q112D+Q4R virus showed a more rapid loss of sensitivity to nevirapine than the RGDA/Q112D virus and the WT virus in both untreated and IFN-β-treated cells (Fig. 8A). In contrast, the RGDA/Q112D and RGDA/Q112D+G94D/G116R viruses delayed the loss of sensitivity to nevirapine compared with the WT virus only in IFN-β-treated cells (Fig. 8A). The effect of IFN-β treatment on the completion of reverse transcription was clearer starting at the time point of 4 h postinfection (Fig. 8B). We observed that while the relative infectivity of the RGDA/Q112D and RGDA/Q112D+G94D/G116R viruses was decreased by IFN-β, that of the WT and the RGDA/Q112D+Q4R viruses was not affected (Fig. 8B).

### The Q4R mutation accelerates initiation of uncoating of the RGDA/Q112D virus.

**FIG 9:**
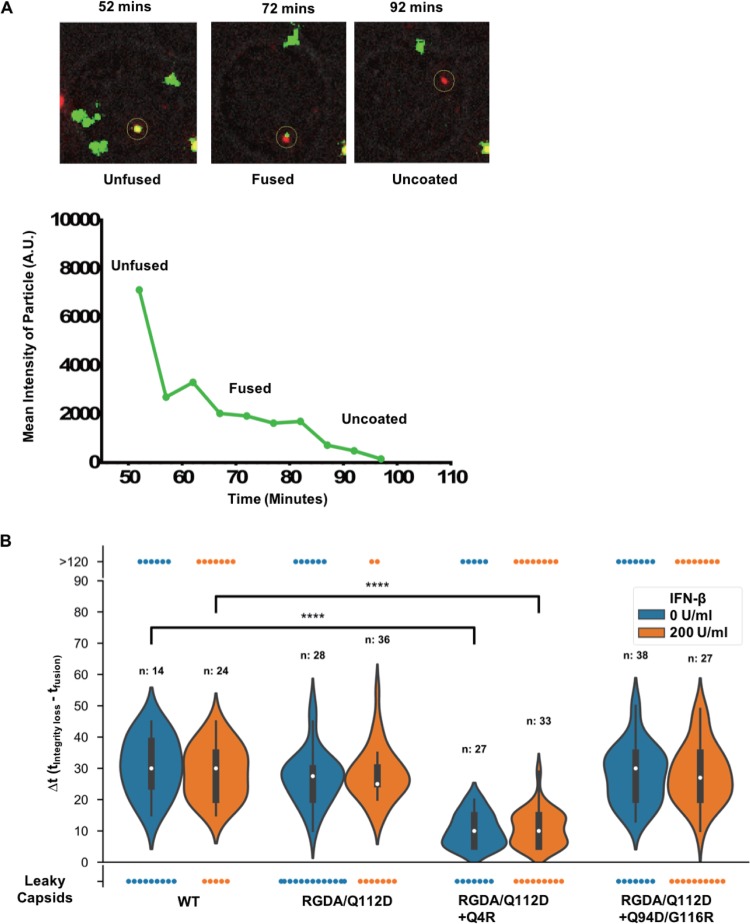
The Q4R mutation accelerates initiation of uncoating of the RGDA/Q112D virus. (A) (Top) Time-lapse images of iGFP-tdTomato-Vpr infection in Jurkat cells (a bright-field cell reference is shown in gray). The GFP signal (green) detected in viral particles, as reported by the tdTomato-Vpr signal (red), was reduced over time until it completely disappeared. (Bottom) Display of the mean particle intensity of the GFP signal (green) of the particle shown in the images at the top. When fusion occurs, there is first a drop of the GFP signal (52.5 min), and when the capsid integrity is compromised, there is a complete loss of the GFP signal (82.5 min). A.U., absorbance units. (B) The difference in the time (*t*) of initiation of uncoating (integrity loss) and the time of fusion (Δ*t*) was calculated for each individual tracked particle. A representation of all iGFP-tdTomato-Vpr particles that fused into the cell is shown. The results for particles that kept HIV-iGFP until the end of the time lapse (>120 min), particles that lost all GFP at fusion (leaky capsid), and particles that showed two signal losses (violin density/probability plots) are shown. For particles with dual drop events, respective medians are shown with white dots, boxes show the interquartile ranges, and vertical lines show the range of all dual drop events. *P* values were determined by the Kruskal-Wallis test followed by Dunn’s multiple comparison. ****, *P *< 0.0001.

We next examined the uncoating kinetics of these CA mutants using a recently developed live-cell-imaging technique (12). This technique utilizes fluid-phase GFP that is trapped inside of the virion to measure fusion and viral core integrity loss. The GFP is located within HIV Gag between MA and CA, with protease sites flanking GFP on both sides (56). During virus maturation, the GFP is cleaved from the Gag within the virions, where a subset becomes trapped within the conical core. As previously reported with this system, we observe the stepwise loss of GFP over time. Upon viral fusion, a majority of the GFP which is not present within the intact core is lost. Later, the fluid-phase GFP within the HIV core is lost when portions of CA are initially shed and the integrity of the viral core (capsid shell) is also lost. While the first drop of the GFP signal reports the timing of viral fusion to the cell, the second and total GFP signal loss indicates the timing of the initiation of uncoating (Fig. 9A). Upon analysis of the kinetics of the initiation of uncoating of hundreds of viral particles, we observed that HIV-iGFP harboring RGDA/Q112D (see Movie S1 in the supplemental material) and RGDA/Q112D+G94D/G116R CA sequences showed the same kinetics of initiation of uncoating as HIV-iGFP harboring WT CA (Fig. 9B; Movie S2). While other behaviors are present, such as long-term GFP retention after fusion or leaky capsids, we previously reported that the early kinetics of the initiation of uncoating ranging from ∼15 min (Movie S1) to ∼45 min (Movie S2) postfusion are linked to infectivity (Fig. 9B) (12, 14).

Strikingly, we observed that RGDA/Q112D+Q4R (Movie S3) had faster kinetics for the initiation of uncoating, a process that has been shown to take place after the first-strand transfer (12, 13). The results obtained from the time-dependent addition of nevirapine (Fig. 8A and B), together with the direct observations from live-cell imaging of viral particles (Fig. 9B), show that RGDA/Q112D+Q4R progresses to initialize reverse transcription earlier and to a faster completion of the process. Interestingly, this acceleration of the initiation of uncoating and the faster initiation and completion of reverse transcription are intrinsic to the RGDA/Q112D+Q4R virus, as the addition of IFN-β had no effect on the kinetics of capsid integrity loss or reverse transcription (Fig. 8). We concluded that the RGDA/Q112D+Q4R mutation accelerated both reverse transcription completion kinetics and the initiation of uncoating relative to those for the RGDA/Q112D and the WT viruses in the presence and absence of IFN-β. In contrast, the RGDA/Q112D virus and the RGDA/Q112D+G94D/G116R virus developed a slower loss of sensitivity to nevirapine than the WT virus, but only in IFN-β-treated cells. There was no change in the kinetics of the initiation of uncoating in the presence and absence of IFN-β.

### The RGDA/Q112D virus obtained IFN-β resistance with the G94D mutation followed by the G116R mutation to compensate for the impaired infectivity.

**FIG 10:**
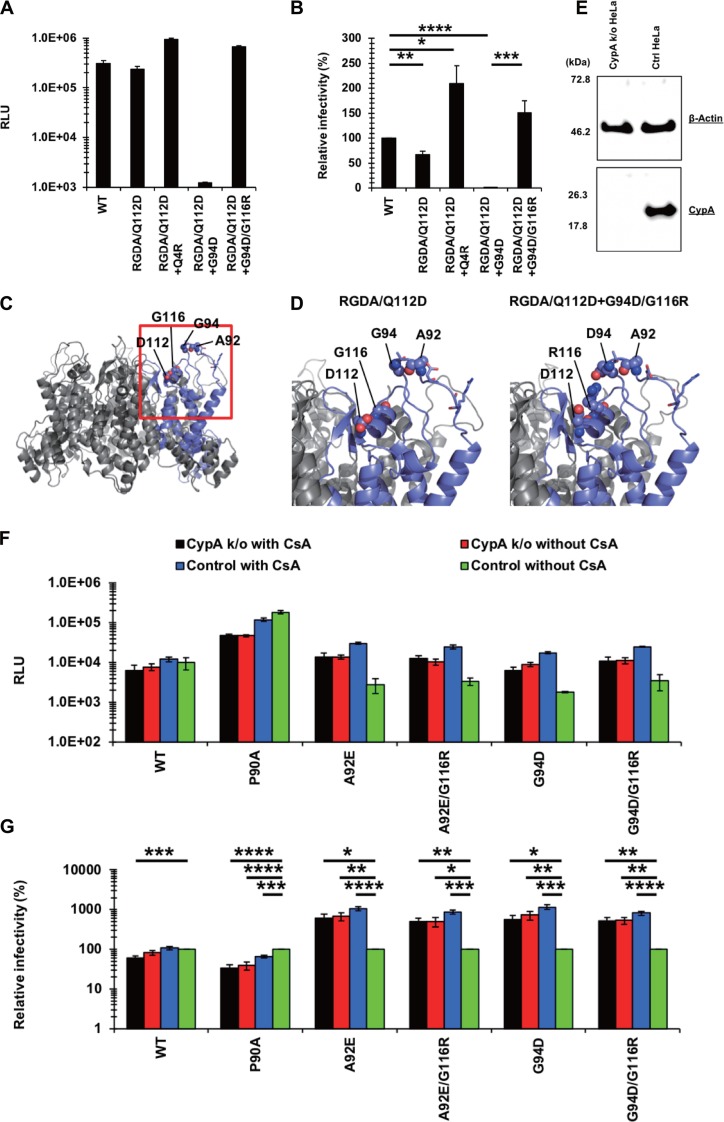

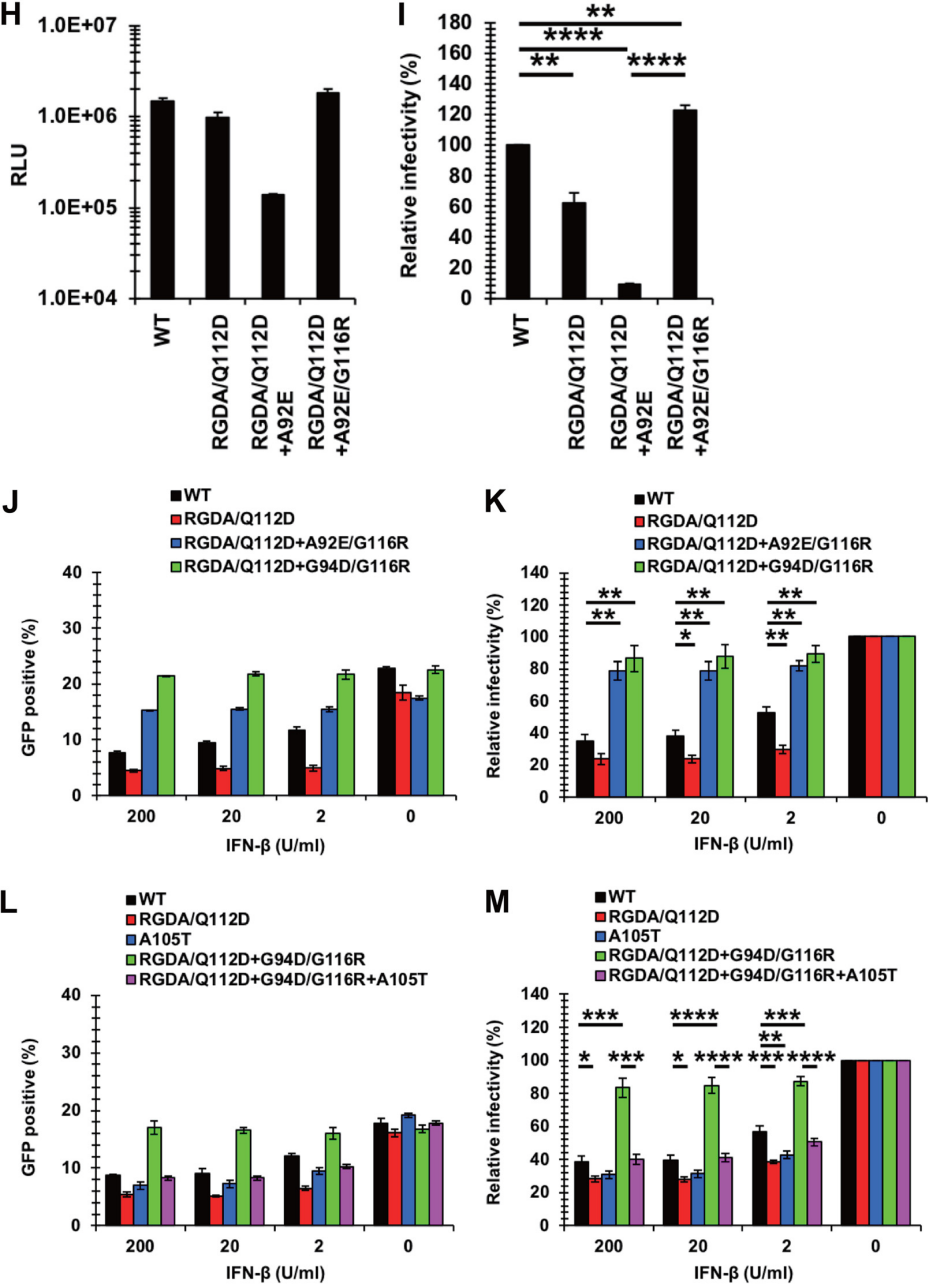
The RGDA/Q112D virus obtained IFN-β resistance with the G94D mutation and then obtained the G116R mutation to compensate for the impaired infectivity. (A) Jurkat cells were infected with reverse transcriptase-normalized VSV-G-pseudotyped HIV-1 isolates encoding the NanoLuc reporter gene. The RLU were determined at 2 days after infection. One representative result of at least three independent experiments is shown, with error bars denoting the standard deviation (SD) of the mean of quadruplicate measurements. (B) The relative infectivity (compared with that of the WT virus [in percent]) was calculated by dividing the RLU of CA mutants by those of the WT virus. The mean from four independent experiments is shown, with error bars denoting the standard error of the mean (SEM). ****, *P *< 0.0001; ***, *P *< 0.001; **, *P *< 0.01; *, *P *< 0.05. (C) A structural model of a hexameric CA mutant of the RGDA/Q112D virus. A single chain is highlighted with a navy ribbon, while the other chains are shown as gray ribbons. The 92nd, 94th, 112th, and 116th residues are shown in sphere representations. The 87th, 88th, 90th, and 93rd residues are drawn as sticks. The 87th, 88th, 90th, 92nd, 93rd, and 94th residues positioned in a loop. The 112th and 116th residues were located in the same face of a helix. (D) Structures around the 94th and 116th residues. The highlighted area corresponds to the area surrounded by the red square in panel B. The G94D/G116R mutations could generate intramolecule salt bridges of R116 with the D94 residue. (E) Expression level of CypA in HeLa cells transduced with the pX459 plasmid targeting the CypA gene. Western blots of cell lysates extracted from unmodified and transduced cells were probed with an anti-CypA antibody (bottom) or an anti-β-actin antibody (top). The positions of the molecular weight markers are shown on the left side. (F) CypA-knockout (CypA k/o) or normal HeLa cells were infected with reverse transcriptase-normalized VSV-G-pseudotyped HIV-1 isolates encoding the luciferase reporter gene in the absence or presence of 2 μM cyclosporine (CsA). The RLU were determined at 2 days after infection. One representative result of at least three independent experiments is shown, with error bars denoting the standard deviation (SD) of the mean of triplicate measurements. (G) The relative infectivity (compared with that for control cells without CsA [in percent]) was calculated by dividing the RLU in CypA k/o or CsA-treated cells by those in control cells without CsA. The mean from five independent experiments is shown, with error bars denoting the standard error of the mean (SEM). ****, *P *< 0.0001; ***, *P *< 0.001; **, *P *< 0.01; *, *P *< 0.05. (H) Jurkat cells were infected with reverse transcriptase-normalized VSV-G-pseudotyped HIV-1 isolates encoding the NanoLuc reporter gene. The RLU were determined at 2 days after infection. One representative result of at least three independent experiments is shown, with error bars denoting the standard deviation (SD) of the mean of quadruplicate measurements. (I) The relative infectivity (compared with that of the WT virus [in percent]) was calculated by dividing the RLU of CA mutants by those of the WT virus. The mean from three independent experiments is shown, with error bars denoting the standard error of the mean (SEM). ****, *P *< 0.0001; **, *P *< 0.01. (J) Jurkat cells were treated with 200, 20, 2, or 0 U per ml of IFN-β for 16 h prior to infection. Cells were infected with VSV-G-pseudotyped HIV-1 isolates encoding GFP. The percentage of GFP-positive cells was determined at 2 days after infection. One representative result of at least three independent experiments is shown, with error bars denoting the standard deviation (SD) of the mean of triplicate measurements. (K) The relative IFN-β sensitivity (compared with that in untreated cells [in percent]) was calculated by dividing the percentage of GFP-positive cells among IFN-β-treated cells by the percentage of GFP-positive cells among untreated cells. The mean from three independent experiments is shown, with error bars denoting the standard error of the mean (SEM). **, *P *< 0.01; *, *P *< 0.05. (L) Jurkat cells were treated with 200, 20, 2, or 0 U per ml of IFN-β for 16 h prior to infection. Cells were infected with VSV-G-pseudotyped HIV-1 isolates encoding GFP. The percentage of GFP-positive cells was determined at 2 days after infection. One representative result of at least three independent experiments is shown, with error bars denoting the standard deviation (SD) of the mean of triplicate measurements. (M) The relative IFN-β sensitivity (compared with that in untreated cells [in percent]) was calculated by dividing the percentage of GFP-positive cells among IFN-β-treated cells by the percentage of GFP-positive cells among untreated cells. The means from five independent experiments are shown, with error bars denoting the standard error of the mean (SEM). ****, *P *< 0.0001; ***, *P *< 0.001; **, *P *< 0.01; *, *P *< 0.05.

As described above (Fig. 2), we identified the RGDA/Q112D+G94D virus and RGDA/Q112D+G94D/G116R virus after adaptation of the RGDA/Q112D virus in IFN-β-treated cells but failed to find the RGDA/Q112D virus with the G116R mutation alone. Moreover, only the G116R mutation was associated with the G94D mutation. We assume that the RGDA/Q112D virus first mutated the G94D position and then acquired the G116R mutation. Our infectivity data support this scenario. The RGDA/Q112D+G94D virus was severely defective, with 0.4% infectivity relative to that of the WT virus (Fig. 10A and B). In contrast, the relative infectivity of the RGDA/Q112D+G94D/G116R virus was 2-fold higher than that of the WT virus (Fig. 10B). We observed that the RGDA/Q112D+Q4R virus had a 1.5-fold higher infectivity than the WT virus.

It should be noted that the replication-defective RGDA/Q112D+G94D virus showed abnormal Gag processing in immunoblots of virions (Fig. 7C, lane 4, marked with asterisks), whereas the RGDA/Q112D+G94D/G116R virus exhibited normal Gag processing (Fig. 7C, lane 5). These observations suggest that the G116R mutation rescued the RGDA/Q112D+G94D virus by compensating for the abnormal Gag processing of the RGDA/Q112D+G94D virus. This idea appears to be supported by structural modeling, in which the 94th residue was located in a loop and it positioned close to the 116th residue (Fig. 10C and D). The R116 residue could form a salt bridge with the D94 residue, suggesting that the G116R mutation rescued the RGDA/Q112D+G94D virus by restoring the interaction between CA amino acids 94 and 116. We also observed that the G94D mutation conferred IFN-β resistance to the WT virus (Fig. 4). These results suggest that the G94D mutation is responsible for conferring IFN-β resistance to the RGDA/Q112D virus, while the G116R mutation is a compensatory mutation to restore the impaired infectivity of the RGDA/Q112D+G94D virus.

Next, we examined the relationship between the A92E CA mutation and the G116R mutation since the NL4-3 A92E CA mutant shares several phenotypes with the NL4-3 G94D CA mutant in terms of sensitivity to CypA and an inability to infect nondividing cells (43, 57–59). Specifically, the infection of the NL4-3 G94D mutant is reduced by endogenous CypA; thus, its infection is rescued by the genetic depletion of CypA or cyclosporine (CsA) treatment in certain cell types, such as HeLa and H9 cells (43, 58–60). Consistent with previous studies, infections of the NL4-3 G94D and A92E viruses were enhanced by CypA knockout or CsA treatment in HeLa cells (Fig. 10E to G). Here we observed that the G116R mutation did not affect the CypA sensitivity of the NL4-3 A92E and G94D viruses. We examined whether the G116R mutation augments the infectivity of the RGDA/Q112D+A92E virus. The RGDA/Q112D+A92E/G116R virus exhibited infectivity significantly higher than that of the RGDA/Q112D+A92E virus (Fig. 10H and I), suggesting that the G116R mutation augmented the infectivity of both the RGDA/Q112D+A92E and the RGDA/Q112D+G94D viruses. Finally, we examined the IFN-β sensitivity of the RGDA/Q112D+A92E/G116R virus and found that the virus was completely resistant even in the highest concentration (200 U per ml) of IFN-β (Fig. 10J and K), demonstrating that the phenotype of the RGDA/Q112D+G94D/G116R virus was shared with that of the RGDA/Q112D+A92E/G116R virus.

Previous studies demonstrated that the impaired infectivity of CsA-dependent CA mutants, including those with the A92E and G94D mutations, in certain cell types was rescued by additional CA mutations, such as P90A and A105T (43, 57, 59). We became interested in how such mutations affected the IFN-β resistance of the RGDA/Q112D+G94D/G116R virus. To this end, we introduced the A105T mutation into the WT and RGDA/Q112D+G94D/G116R viruses. Consistent with the previous observation in THP-1 cells (10), the A105T virus showed higher IFN-β sensitivity than the WT virus in Jurkat cells (Fig. 10L and M). We observed a statistically significant difference between the WT and A105T viruses in cells treated with 2 U/ml of IFN-β. Interestingly, the RGDA/Q112D+G94D/G116R+A105T virus exhibited enhanced IFN-β sensitivity compared with the RGDA/Q112D+G94D/G116R virus. This result suggested that the prominent resistance of the RGDA/Q112D+G94D/G116R virus to IFN-β-mediated restriction was on a delicate balance; thus, just one CA mutation would reverse this phenotype.

We concluded that the G94D mutation was responsible for conferring IFN-β resistance to the RGDA/Q112D virus, whose impaired infectivity was rescued by the G116R mutation.

## DISCUSSION

**FIG 11:**
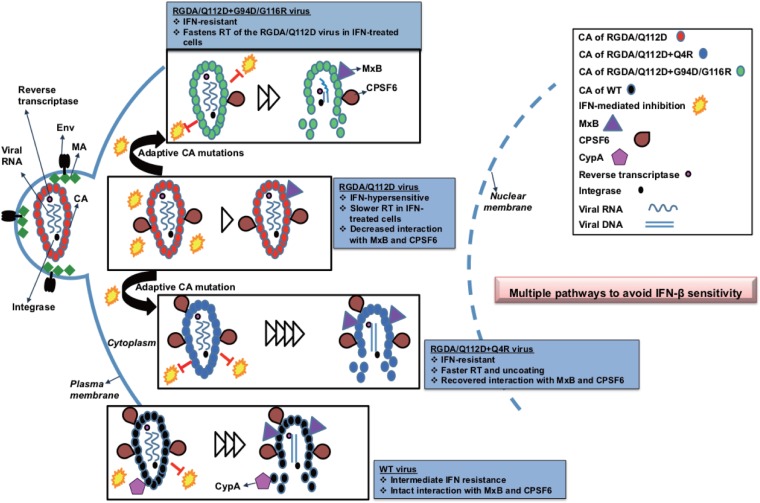
Multiple pathways to avoid IFN sensitivity of HIV-1 by mutations in the capsid. An IFN-hypersensitive CA mutant RGDA/Q112D virus (red) evolved to be IFN resistant by acquiring additional Q4R or G94D/G116R mutations. The IFN-resistant RGDA/Q112D+Q4R virus (blue) shows accelerated kinetics of reverse transcription (RT) and a faster initiation of uncoating in both the presence and the absence of IFN. The virus shows recovered interactions with MxB and CPSF6. Another IFN-resistant virus, the RGDA/Q112D+G94D/G116R virus (green), accelerates the kinetics of reverse transcription to a smaller degree only in the presence of IFN. The virus showed a degree of interaction with MxB similar to that of the RGDA/Q112D virus and a weaker interaction with CPSF6 than the RGDA/Q112D virus. The WT virus (black) showed intermediate IFN resistance and intact interactions with MxB, CypA, and CPSF6.

We have demonstrated that HIV-1 can utilize multiple mutational pathways to overcome the capsid-targeting antiviral activities induced by type I interferons in T cells. A unique HIV-1 CA mutant virus which is unusually hypersensitive to IFN-β was used to study the capsid-targeting inhibitory effects of type I IFN. Adaptation of this mutant revealed two mutational pathways allowing viral escape. The single Q4R mutation or the double substitutions G94D/G116R in CA which emerged in this adaptation conferred IFN-β resistance to the IFN-β-hypersensitive RGDA/Q112D mutant virus. Notably, the Q4R mutation drastically altered multiple CA properties, including the acceleration of the kinetics of the completion of reverse transcription and the initiation of uncoating relative to those in WT HIV, in the presence or absence of IFN-β (Fig. 8 and 9). In contrast, the IFN-β resistance obtained by the G94D/G116R mutation was clearly achieved through a different mechanism because the kinetics of the completion of reverse transcription were delayed relative to those in the WT virus only in the presence of IFN-β (Fig. 8). It is notable that the kinetics of the completion of reverse transcription by the RGDA/Q112D virus were delayed relative to those in the WT only in the presence of IFN-β. The Q4R and G94D/G116R mutations also impacted the interaction with the known cellular factors CPSF6 and MxB in a different manner (Fig. 6 and 7). While the sensitivity of the RGDA/Q112D virus to MxB and CPSF6-358 was enhanced only by the Q4R mutation, neither the Q4R mutation nor the G94D/G116R mutations allowed the recovery of the deficient CypA binding of the RGDA/Q112D virus. These observations suggest the limited IFN-β resistance contribution of known capsid-binding host factors to this IFN-β-hypersensitive mutant. Overall, these observations reveal that HIV-1 can utilize multiple mechanistic pathways to overcome IFN-β-mediated restriction (Fig. 11).

In this study, we utilized the RGDA/Q112D virus, which is hypersensitive to IFN-β, to investigate how the HIV-1 capsid can evade type I IFN-mediated restriction. We identified two different pathways of resistance which involve either the Q4R change or the G94D/G116R changes in the CA after adaptation of the RGDA/Q112D virus in IFN-β-treated cells (Fig. 2). We searched for the presence of these substitutions in the primary HIV-1 isolates and found that the 4th (Q4) and 94th (G94) amino acids are highly conserved, while the 116th (G116) amino acid is relatively polymorphic. It is reasonable to speculate that the Q4R and G94D mutations are specifically associated with the RGDA/Q112D virus. Importantly, these CA mutations were sufficient to reverse the IFN-β hypersensitivity of the RGDA/Q112D parental virus. The impact of these mutations allowing IFN-β resistance was obvious, as the RGDA/Q112D viruses harboring these mutations were even more IFN-β resistant than the WT virus in a single-cycle infection assay (Fig. 3A and B). Interestingly, the effect of the G94D/G116R mutations was reproduced even in the WT virus (Fig. 4). Thus, the influence of the G94D/G116R mutations on IFN-β resistance was not specific to the RGDA/Q112D virus. These findings further support the hypothesis that HIV-1 isolates with the Q4R and G94D/G116R mutations have different mechanisms to overcome their sensitivity to type I IFNs.

CPSF6 is a host factor that is capable of modulating the type I IFN sensitivity of HIV-1 since CPSF6 binding-deficient CA mutant N74D virus was shown to be hypersensitive to type I IFN in myeloid cell line THP-1 cells (10). We observed that the IFN-β-hypersensitive RGDA/Q112D virus was less sensitive to CPSF6-358 inhibition than the WT virus and that this change was restored by the Q4R mutation to a level of that of the WT virus (Fig. 7A and B). These observations are consistent with the suggested link between CPSF6 binding and IFN-β sensitivity. The opposite effect on the CPSF6-358 sensitivity of the RGDA/Q112D virus was observed with the other adaptive change, the G94D/G116R mutations. It appeared that the RGDA/Q112D virus acquired two variants that differently changed CPSF6 binding. Both of these adapted variants were more resistant to IFN-β (Fig. 3A and B), suggesting that CPSF6 is not involved in the development of our observed resistance to type I IFNs in T cell lines, such as Jurkat cells.

CypA binding is another factor that possibly affects the type I IFN sensitivity of HIV-1, since CypA binding-deficient CA mutant P90A was shown to be hypersensitive to type I IFN in THP-1 cells (10). The RGDA/Q112D virus lost its CypA binding ability and exhibited enhanced IFN-β sensitivity, consistent with a potential role of CypA in reducing sensitivity to type I IFNs. Our results demonstrate that neither of the CA mutations that arose during adaptation of the RGDA/Q112D virus in IFN-β-treated cells restored the CypA binding of the RGDA/Q112D virus (Fig. 7). These findings suggest that CypA binding is dispensable for the IFN-β resistance of HIV-1 in T cells. Bulli et al. showed that depletion of the gene for CPSF6 or CypA in THP-1 cells did not overtly affect the type I IFN sensitivity of the WT virus (10), a finding which supports our hypothesis. Considering the link between CypA and the type I IFN sensitivity of HIV-1, Bulli et al. proposed that cyclophilins other than CypA are involved in HIV-1 sensitivity to type I IFN, as CsA treatment of THP-1 cells rescued the impaired infectivity of the P90A virus in IFN-α-treated cells (10). It is possible that these cyclophilins are involved in IFN-β-mediated inhibition of the RGDA/Q112D virus in T cells. Future studies need to be performed to address this hypothesis.

The early steps of HIV-1 infection are blocked by a type I IFN-inducible factor, MxB, whose antiviral effect is influenced by viral CA (27, 30). Despite its hypersensitivity to type I IFN, the RGDA/Q112D virus was, paradoxically, resistant to MxB (Fig. 1, 2, and 6). A similar phenotype was observed with N74D and P90A CA mutants in terms of type I IFN hypersensitivity and resistance to MxB (10, 27, 30). The RGDA/Q112D virus was even more resistant to MxB than the N74D virus in our experimental settings (Fig. 6F). The RGDA/Q112D virus evolved to be IFN-β resistant, with two distinct variants differently affecting the sensitivity of the RGDA/Q112D virus to MxB. The G94D/G116R mutations do not change the sensitivity of the RGDA/Q112D virus to MxB (Fig. 6F). In contrast, the Q4R mutation enhanced the sensitivity of the RGDA/Q112D virus to MxB, despite the higher IFN-β resistance of the RGDA/Q112D+Q4R virus (Fig. 3 and 6F). These results suggest that MxB does not contribute to the IFN-β sensitivity of the RGDA/Q112D virus in T cells, and these findings are similar to those of previous studies that suggested a limited role of MxB in restriction mediated by type I IFN in myeloid THP-1 cells (10, 61). During preparation of the manuscript for this article, Xu et al. argued that the limited role of MxB reported previously was due to experimental conditions with HIV-1 carrying the VSV G protein (62). It should be noted that our results were obtained with replication-competent viruses harboring HIV-1 Env (Fig. 3 and 4).

While the observations presented above suggest that the evasion of IFN-β-mediated restriction in this system is not a consequence of an altered interplay between CA and known host factors, restriction mediated through a CA function could take place through multiple mechanisms. Although there has been much discussion about the intact core protecting the viral nucleic acids from being recognized (and potentially degraded) by host factors, the repeating array of CA presented within the HIV fullerene core can also function as a pathogen-associated molecular pattern (PAMP). As an example, it has been shown that core recognition by TRIM5α can induce an antiviral state (63). Bulli et al. also proposed that inappropriate uncoating kinetics might account for the higher type I IFN sensitivity of the N74D virus (10). We propose that one potential mechanism of evasion of a type I IFN-induced antiviral activity targeting CA determinants could be an accelerated rate of uncoating. The acceleration of the process of uncoating would also require the acceleration of other aspects of the early HIV life cycle. A consensus is emerging that the process of uncoating, especially the initiation of this process, is dependent on reverse transcription (13, 14). Likely, the process must advance beyond the first-strand transfer before the initiation of uncoating can proceed. The virus could facilitate the evasion of IFN-β sensitivity that targets CA by accelerating the process of uncoating. Based on our previous work demonstrating a connection between the progression of the steps of reverse transcription and the initiation of uncoating, the pathway of IFN-β escape could be mediated directly through a change of the stability of the core or indirectly through the acceleration of reverse transcription. For instance, the N74D and E45A mutations appear to directly decrease the rate of uncoating without any impact on the kinetics of reverse transcription (12, 54). Interestingly, the virus acquiring the Q4R mutation took the indirect route to evade enhanced IFN-β sensitivity by accelerating the process of reverse transcription, which subsequently accelerated the initiation of uncoating. This Q4R mutation in the RGDA/Q112D derivative changed the intrinsic properties of the virus to accelerate reverse transcription and the initiation of uncoating in the presence or absence of IFN-β (Fig. 8A and 9B). This phenotype further validates the model that uncoating can be modulated by reverse transcription. The phenotype of the virus with the Q4R mutation shows us that accelerated reverse transcription and initiation of uncoating are clearly a potential mechanism to avoid IFN-β sensitivity targeting the core as a PAMP.

The other G94D/G116R mutation overcoming IFN-β sensitivity clearly utilizes a distinct mechanism. Considering the roles of the G94D/G116R mutations for the RGDA/Q112D virus, we speculate that the G94D mutation is responsible for conferring IFN-β resistance to the RGDA/Q112D virus, whereas the G116R mutation is a compensatory mutation to rescue the infectivity of the RGDA/Q112D+G94D virus (Fig. 10). This evolution of the RGDA/Q112D virus in IFN-β-treated cells is of interest since the G94D mutation is detrimental to the infectivity of the RGDA/Q112D virus (Fig. 10A and B). This result may imply that the RGDA/Q112D virus was under strong evolutionary pressure to select for the G94D mutation to facilitate IFN-β resistance, even if the mutation is harmful for its infectivity. This is consistent with IFN-β sensitivity requiring some interaction with position 94 in CA.

In this study, we demonstrate that two types of CA mutations were sufficient to confer IFN-β resistance to the RGDA/Q112D virus. However, the frequency of each type of CA mutation in the adapted viruses was not very high (only 30%, 5%, and 10% for the RGDA/Q112D+Q4R, RGDA/Q112D+G94D, and RGDA/Q112D+G94D/G116R mutations, respectively). One possible reason for this is that mutations in other regions of the HIV-1 genome, rather than CA, might account for the IFN-β resistance of the adapted viruses. In addition to the aforementioned mutations in CA, we have identified an A15T mutation in Nef of IFN-β-resistant viruses that warrants further investigation (data not shown). We are interested in why the noninfectious RGDA/Q112D+G94D virus represented 5% of all viruses (Fig. 2). It may be reasonable to speculate that, in addition to the G116R mutation in CA (Fig. 10A and B), Nef A15T might compensate for the impaired infectivity of the RGDA/Q112D+G94D virus. The impact of the Nef A15T mutation needs to be elucidated in future studies. Alternatively, our data might include sequences of replication-defective viruses. A limitation of this study is that we used an established series of CA mutations (RGDA/Q112D virus) as a starting viral backbone, since we previously aimed at making an HIV-1 clone with a high level of resistance to macaque TRIMCyp (40). This virus harbors CA mutations that are not present in circulating HIV-1 strains. It is reasonable to assume that the Q4R mutation arising during adaptation in IFN-β-treated cells might be specifically related to the RGDA/Q112D virus, whereas the effect of the G94D/G116R mutations was reproduced even in the WT virus. Our findings portray the tremendous well-known plasticity of HIV-1, which can be one of the biggest barriers in therapy.

The present study shed light on the evolution of an IFN-β-hypersensitive CA mutant in Jurkat cells, which is a line of T cells, the major target cells of HIV-1 infection. Given that ISG induction and an antiviral effect upon IFN treatment are more evident in myeloid cells than in T cells (64, 65), THP-1 cells are widely used for investigating the IFN sensitivity of HIV-1 (10, 61). While we were able to demonstrate that the Q4R and G94D/G116R mutations conferred partial IFN-β resistance to the RGDA/Q112D virus in THP-1 cells (Fig. 3E and F), further studies will be required to elucidate whether similar pathways are utilized to evade IFN-mediated restriction in cells of myeloid cell origin.

In this study, we studied the sensitivity of CA mutants to an already established restriction mediated by IFNs. The production of IFNs in the target cells as a result of viral challenge with each CA mutant is another topic. HIV-1 DNA in the cytosol is sensed by intracellular DNA sensors, including gamma interferon-inducible protein 16 (IFI16) and cyclic GMP-AMP synthase (cGAS), in cells of myeloid cell origin (66, 67). In the case of HIV-1 DNA recognition in T cells, Berg et al. showed that HIV-1 DNA does not induce ISGs in activated CD4^+^ T cells, regardless of detectable expression levels of DNA sensors in these cells (68). Consistent with this finding, we failed to detect IFNs in the culture supernatants of Jurkat cells infected with either WT or RGDA/Q112D viruses (data not shown). It will be interesting to test whether our novel CA mutants are capable of inducing IFNs in cells of myeloid cell origin in future studies.

Overall, the data generated in this study demonstrate that the IFN-β-hypersensitive RGDA/Q112D virus can evolve to be IFN-β resistant with two distinct variants: a virus with the single Q4R mutation in CA or a virus with the double substitutions G94D/G116R in CA. The Q4R mutation changes the CA properties of the RGDA/Q112D virus, including sensitivity to MxB and CPSF6-358, compared with those of the RGDA/Q112D virus. This RGDA/Q112D+Q4R mutation accelerates reverse transcription kinetics and the timing of the initiation of uncoating in an IFN-β-independent manner. In contrast, the RGDA/Q112D+G94D/G116R mutation induces a change in reverse transcription only in the presence of IFN-β, with the kinetics of reverse transcription being slower in the RGDA/Q112D+G94D/G116R virus than in the WT virus. Moreover, the RGDA/Q112D+G94D/Q116R mutation did not alter the kinetics of the initiation of uncoating relative to those in the WT virus. Our findings reveal that HIV-1 is able to select multiple independent pathways in order to avoid restriction mediated by type I IFN by generating mutations in CA.

## MATERIALS AND METHODS

### Plasmid DNAs.

Env-deleted molecular clones encoding the CA of the NL4-3 strain of HIV-1 carrying either the GFP gene (pMSMnG) (69) or the luciferase gene (pNL4-3.Luc.R^−^E^−^; NIH AIDS Research and Reference Reagent Program) in place of the *nef* gene were used in the present study. We also used pBru3oriΔEnv-luc2 (70, 71) and pBru3oriΔEnv-NanoLuc plasmids, in which the BssHII/ApaI fragments were replaced with the corresponding fragment of pNL4-3 plasmids. To generate replication-competent virus, we used the pNL4-3 plasmid (72) and the pNL-vifS plasmid, which harbors the entire *vif* gene of the simian immunodeficiency virus SIVmac239 in place of the NL4-3 *vif* gene and which was previously termed pNL-SVR (36). Various CA mutations were introduced into these clones using standard cloning procedures as described previously (57). The DNA plasmid encoding the vesicular stomatitis virus G glycoprotein (VSV-G) (pMD2G) was described previously (73). HIV-Gag-iGFPΔEnv and psPAX2 were used as described by Mamede et al. (12), and the CA sequences of both plasmids were mutated: RGDA/Q112D, RGDA/Q112D+Q4R, and RGDA/Q112D+G94D/G116R. We verified all PCR-amplified regions of the plasmids by Sanger sequencing. To pseudotype the virions that were used for live-cell imaging, we used pCMV-VSV-G as previously described (12, 14). ptdTomato-Vpr had the GFP sequence swapped from pGFP-Vpr and was previously described (74, 75).

### Cell culture.

HEK293T cells (ATCC) and HeLa cells (ATCC) were cultured in Dulbecco’s modified Eagle’s medium supplemented with 10% fetal bovine serum (FBS) and 1× penicillin-streptomycin (P/S). Immortalized suspension cells (MT4 [ATCC], THP-1 [ATCC], and Jurkat Luc knockdown [Jurkat Luc(k/d)] cells stably expressing short hairpin RNA against luciferase [kindly provided by Jeremy Luban {76}]) were cultured in RPMI supplemented with 10% FBS and 1× P/S.

### Viruses.

All HIV-1 isolates were generated by transfecting HEK293T cells using polyethylenimine (PEI; PolySciences). Recombinant SeVs expressing MxB (77), CM TRIMCyp (78), CM TRIM5α without the SPRY domain (CM SPRY^−^) (79), murine CPSF6-358, and CPSF6-358-FG321/322AA (80) were recovered as previously described (81). The viruses that were passaged a second time in embryonated chicken eggs were used as the stock for all experiments.

### Infection.

For the single-round infection assay, 2.5 × 10^5^ per ml Jurkat, MT4, and THP-1 cells were treated with 0, 2, 20, or 200 U per ml of recombinant IFN-β (catalog number 300-02BC; PeproTech) for 16 h prior to infection. Cells were infected with VSV-G-pseudotyped HIV-1 isolates encoding GFP. Note that each concentration of IFN-β was maintained in the cell cultures during virus infection. Virus infectivity was determined at 2 days after infection by measuring the GFP-positive cells using a FACSCalibur flow cytometer (BD Biosciences) or an EC800 cell analyzer (Sony). For multiple-round infection assays, 2.5 × 10^5^ per ml Jurkat cells were treated with 100 U per ml of IFN-β or left untreated for 16 h prior to infection. To compare the replication capability of the CA mutants, we normalized the input virus by the amounts of reverse transcriptase in the supernatant, as determined by a SYBR green PCR-enhanced reverse transcription (SG-PERT) assay previously described (82). NL4-3 viruses normalized to 1,000 pg per ml were added to the cells. After spinoculation at 1,200 × *g* for 60 min, the cells were washed twice and resuspended in fresh medium with or without 100 U per ml of IFN-β. Half of the culture medium was replaced with fresh medium with or without 100 U per ml of IFN-β every 3 days, and the concentration of reverse transcriptase in the culture supernatant was quantified by the SG-PERT assay. To compare the relative infectivity of the CA mutants, Jurkat cells were infected with reverse transcriptase-normalized HIV-1 isolates encoding the NanoLuc reporter gene. The cells were lysed at 2 days after infection, and the relative luciferase units (RLU) were measured using the Nano-Glo luciferase assay reagent (Promega) on a luminometer. The relative infectivity of the CA mutants (compared with that of the WT virus [in percent]) was calculated by dividing the RLU of the CA mutants by those of the WT virus. To test the sensitivity of the CA mutants to MxB, CPSF6-358, or CM TRIMCyp, MT4 cells were infected with SeVs expressing these host factors for 6 h at a multiplicity of infection (MOI) of 10. Cells were superinfected with reverse transcriptase-normalized VSV-G-pseudotyped HIV-1 isolates encoding the luciferase reporter gene. The RLU were determined at 2 days after infection using the Bright-Glo luciferase assay reagent (Promega) on a luminometer. The degree of sensitivity to MxB, CPSF6-358, or CM TRIMCyp was calculated by dividing the RLU of each virus in the presence of host factors by those in the presence of CPSF6-358-FG321/322AA or CM SPRY^−^. CypA-knockout (CypA k/o) or control HeLa cells were infected with reverse transcriptase-normalized VSV-G-pseudotyped viruses encoding the luciferase reporter gene by spinoculation (1,200 × *g* for 30 min) in the presence of 20 μg per ml of DEAE-dextran. The cells were lysed at 2 days after infection with a cell culture lysis reagent (Promega) and used to measure luciferase activity with a luciferase assay kit (Promega) on a luminometer. If noted, the cells were cultured in the presence of 2 μM cyclosporine (CsA; Sigma-Aldrich).

### Adaptation of the RGDA/Q112D virus in IFN-β-treated Jurkat cells.

Prior to infection, 3 × 10^5^ Jurkat cells were treated with 100 U per ml of IFN-β or left untreated for 6 h at 37°C. The cells were then infected with 100 ng (p24) of the NL-VifS virus encoding the RGDA/Q112D mutation. After incubation for 2 h, the cells were washed and resuspended in fresh medium with or without 100 U per ml of IFN-β. The cells were maintained with culture medium with or without 100 U per ml of IFN-β throughout the experiment. Culture supernatants were periodically collected, and the viral titers were measured using a p24 RetroTek antigen enzyme-linked immunosorbent assay (ELISA) kit (ZeptoMetrix) according to the manufacturer’s instructions.

### Sequencing analysis of the viral genome.

Jurkat cells infected with the RGDA/Q112D virus were harvested for DNA extraction at 92 days after infection. The genomic DNA was extracted from infected cells with a DNeasy blood and tissue kit (Qiagen) according to the manufacturer’s instructions. A CA fragment was amplified from genomic DNA with PrimeSTAR GXL DNA polymerase (TaKaRa) using forward primer 5′-GCCAGAGGAGATCTCTCGACGCAGG-3′ and reverse primer 5′-TAGGGGCCCTGCAATTTTTGGCTATGTGCCCTTC-3′. The PCR products were cloned into the pCR2.1-TOPO vector (Thermo), and 20 clones were subjected to sequencing analysis with 3130xl genetic analyzers (Applied Biosystems).

### Western blot analysis.

Pelleted cells were resuspended in 1× NuPAGE LDS sample buffer (Thermo) containing 2% β-mercaptoethanol. As an indicator of type I IFN-stimulated gene (ISG) induction, expression of the interferon-stimulated 15-kDa protein (ISG15) was examined with Western blot analysis using a rabbit anti-ISG15 polyclonal antibody (Cell Signaling Technology), followed by horseradish peroxidase (HRP)-conjugated protein A (GE Healthcare). Expression of MxB in Jurkat cells was evaluated by Western blotting using a goat anti-MxB polyclonal antibody (Santa Cruz Biotech), followed by an HRP-conjugated donkey anti-goat IgG antibody (Santa Cruz Biotech). The expression of HA-tagged host factors in SeV-infected MT4 cells was confirmed by Western blotting using a rat anti-HA monoclonal antibody (Roche Diagnostics) followed by an HRP-conjugated goat anti-rat IgG antibody (American Qualex). Chemiluminescence was detected using the Chemi-Lumi One Ultra reagent (Nacalai Tesque) according to the manufacturer’s instruction.

### Expression of endogenous MxB upon SeV infection.

MT4 cells were infected with SeV expressing CPSF6-358-FG321/322AA-HA at an MOI of 10. After 6 h of incubation, we treated SeV-infected or uninfected MT4 cells with 200 or 0 U per ml of IFN-β. After 16 h of incubation, the expression level of endogenous MxB was examined by Western blotting.

### Virion incorporation assay.

HEK293T cells were cotransfected with pNL4-3 plasmids harboring the CA mutations along with the pCEP4 mammalian expression vector, encoding HA-tagged human CypA, as described previously with slight modifications (40). Cells were cultured in the presence of 2 μM CsA when described. Reverse transcriptase-normalized viruses in 900 μl of culture medium were layered onto 500 μl of 20% sucrose in phosphate-buffered saline (PBS) and centrifuged at 20,000 × *g* for 2 h at 4°C. Pelleted virions were resuspended in 1× NuPAGE LDS sample buffer (Thermo) containing 2% β-mercaptoethanol, and the lysed virions were subjected to Western blotting. The HA-tagged CypA and p24 CA proteins were probed with a rat anti-HA monoclonal antibody (Roche Diagnostics) and mouse anti-p24 antibody (Abcam), respectively.

### Structure modeling of CA mutants.

Hexameric CA structural models of each CA mutant were constructed using the Modeller program (83) based on a hexameric CA crystal structure (PDB accession number 3H4E) (84). The figures with the model structures were generated with the PyMOL program (https://pymol.org/).

### Time-of-addition assay.

Time-of-addition experiments were performed by infecting Jurkat cells with VSV-G-pseudotyped viruses encoding the GFP reporter gene by spinoculation (1,200 × *g* for 30 min) in the presence of 20 μg per ml of DEAE-dextran. Cells were washed and cultured in fresh medium. Nevirapine at 2 μM (final concentration; Sigma-Aldrich) was added at the time points indicated above. Viral infectivity was measured by determining the percentage of GFP-positive cells 2 days after infection using the EC800 cell analyzer (Sony).

### Quantification of second-strand transfer products.

Jurkat cells pretreated with 0 or 200 U per ml of IFN-β were infected with reverse transcriptase-normalized VSV-G-pseudotyped viruses encoding the GFP reporter gene by spinoculation (1,200 × *g* for 30 min) in the presence of 20 μg per ml of DEAE-dextran. Viruses were treated with Turbo DNase (Thermo Fisher Scientific) prior to infection. Cells were washed and cultured in fresh medium. The genomic DNA was extracted from cells at 2, 4, and 6 h after infection with a DNeasy blood and tissue kit (Qiagen). Second-strand transfer products were quantified using a TaqMan universal master mix (Thermo Fisher Scientific) and the following primers and probe: primers 2ndTF-F (5′-TTTTAGTCAGTGTGGAAAATCTGTAGC-3′) and 2ndTF-R (5′-TACTCACCAGTCGCCGCC-3′) and probe 2ndTF-Probe (5′-FAM-TCGACGCAGGACTCGGCTTGCT-TAMRA-3′, where FAM is 6-carboxyfluorescein and TAMRA is 6-carboxytetramethylrhodamine) (85). The PCR conditions were 95°C for 10 min, followed by 40 cycles of 95°C for 15 s and 60°C for 1 min. The fluorescent signals were detected with a 7500 Fast real-time PCR system (Thermo Fisher Scientific). A standard curve was generated using quantities of plasmids ranging from 1 × 10^1^ to 1.0 × 10^7^ copies per reaction mixture. Infected cells treated with 5 μM nevirapine served as a negative control.

### Uncoating assay.

A DeltaVision wide-field microscope (GE Life Sciences) equipped with an electron-multiplying charge-coupled device (EM CCD) camera and a solid-state illumination (solid-state illumination light-emitting diode) light path was used to acquire time-lapse fluorescent snapshots of HIV-iGFP/tdTomato-Vpr viruses infecting Jurkat cells plated in Delta T culture dishes (Bioptechs) that were coated with poly-l-lysine (Sigma) according to the supplier’s instructions. The cells were kept in a 37°C heated chamber, together with a blood gas mixture (5% CO_2_, 20% oxygen), throughout the imaging process. The cells were incubated with RPMI without phenol red with 10% FBS, l-glutamine, and minimal essential medium-nonessential amino acids. All infections were done with polybrene at a concentration of 5 μg/ml. The z-stacking spacing was set to 0.5 μm with a total of 12-μm *z*-axis imaging for the fluorescence snapshots, and a single z reference image was taken in bright-field mode for cell edge identification. A total of 42 visit points were acquired per condition (cells incubated with 0 or 200 U per ml of IFN-β, 16 h of preinfection) and per viral mutation. The nominal magnification was ×60 (with a 1.42-numerical-aperture lens from Olympus) for all experiments.

### Live imaging analysis.

z-stacks were deconvolved and z-projected using SoftWorx software (GE Life Sciences), before individual tdTomato-Vpr particle tracking over time was performed using Fiji/ImageJ software (NIH). The mean intensities of HIV-iGFP were automatically measured on the same *x-y* coordinates to which the Vpr particle was tracked. Centered particle video recordings were automatically generated by in-house-made Python scripts using the Pims (http://soft-matter.github.io/pims/ ) and Matplotlib libraries, with the data being analyzed and exported from Fiji/ImageJ software as described by Mamede et al. (12).

### Fixed imaging.

Jurkat cells were treated overnight with or without 200 U per ml of IFN-β. The cells were then allowed to settle on poly-l-lysine-treated glass coverslips for 2 h at 37°C in a 5% CO_2_ incubator. The cells were then carefully washed with PBS and readily fixed with 3.7% formaldehyde (final concentration) in piperazine-*N*,*N*′-bis(2-ethanesulfonic acid) (PIPES) buffer for 5 min, followed by three PBS washes. Cells were permeabilized for 10 min in blocking buffer (10% normal donkey serum, 0.01% NaN_3_, 0.1% Triton X-100), followed by incubation with ISG15 antibody from Abcam (catalog number ab133346) at 1:200 for 1 h at room temperature. The monoclonal antibodies were washed three times with PBS and stained with anti-rabbit Rhodamine Red-X antibodies (1:500) and Hoechst dye (1:25,000) for 30 min. Secondary antibodies were washed three times with PBS and mounted on slides using a Vectashield wet mount. Cells were imaged in a Deltavision wide-field microscope.

### Statistical analysis.

Differences in infectivity between different conditions (e.g., between IFN-β-treated and untreated cells, between cells expressing host factors and cells expressing the control molecule, between the WT and the CA mutants) were examined by an unpaired Student's *t* test. *P* values of 0.05 or less were considered statistically significant. The comparisons of the data obtained from the live-cell imaging were done by considering the data to be nonnormally distributed. The Kruskal-Wallis and Dunn’s multiple comparison statistical tests were performed.

## Supplementary Material

Supplemental file 1

Supplemental file 2

Supplemental file 3

Supplemental file 4
