# Supplementary material for: Multiple Pathways To Avoid Beta Interferon Sensitivity of HIV-1 by Mutations in Capsid
Source: J Virol. 2019 Nov 13;93(23):e00986-19. doi: 10.1128/JVI.00986-19 (PMC6854511; doi:10.1128/JVI.00986-19)
Supplement: Supplemental file 1 [file JVI.00986-19-s0004.pdf]

Supplemental Material

S1 Movie. Time-lapse image of a centered, dual-labeled HIV RGDA/Q112D particle challenging a Jurkat cell.

The mean intensity of the tracked particle is plotted (y axis) for iGFP (green) and tdTomato-Vpr (red) signals (Top Left). The first drop of GFP signal occurs with fusion and a second total drop occurs when the capsid integrity is lost (at ~18 minutes after fusion). The Right panel shows the signal for iGFP (green), tdTomato-Vpr (red), and bright field signal for cell reference (gray). Particle box shows the signal of GFP (green) and tdTomato-Vpr (red) over time. (Bottom Left) Smaller boxes show the signal (gray) of each individual fluorescence measurement as labeled and mixed signal for iGFP (green) and tdTomato-Vpr (red).

S2 Movie. Time-lapse image of a centered, dual-labeled HIV wild-type particle challenging a Jurkat cell.

The mean intensity of the tracked particle is plotted (y axis) for iGFP (green) and tdTomato-Vpr (red) signals (Top Left). The first drop of GFP signal occurs with fusion and a second total drop occurs when the capsid integrity is lost (at ~45 minutes after fusion). The Right panel shows the signal for iGFP (green), tdTomato-Vpr (red), and bright field signal for cell reference (gray). Particle box shows the signal of GFP (green) and tdTomato-Vpr (red) over time. (Bottom Left) Smaller boxes show the signal (gray) of each individual fluorescence measurement as labeled and mixed signal for iGFP (green) and tdTomato-Vpr (red).

S3 Movie. Time-lapse image of a centered, dual-labeled HIV RGDA/Q112D+Q4R particle challenging a Jurkat cell.

The mean intensity of the tracked particle is plotted (y axis) for iGFP (green) and tdTomato-Vpr (red) signals (Top Left). The first drop of GFP signal occurs with fusion and a second total drop occurs when the capsid integrity is lost (at ~10 minutes after fusion). The Right panel shows the signal for iGFP (green), tdTomato-Vpr (red), and bright field signal for cell reference (gray). Particle box shows the signal of GFP (green) and tdTomato-Vpr (red) over time. (Bottom Left) Smaller boxes show the signal (gray) of each individual fluorescence measurement as labeled and mixed signal for iGFP (green) and tdTomato-Vpr (red).
